# Sex-specific impacts of prenatal bisphenol A exposure on genes associated with cortical development, social behaviors, and autism in the offspring’s prefrontal cortex

**DOI:** 10.1186/s13293-024-00614-2

**Published:** 2024-05-15

**Authors:** Songphon Kanlayaprasit, Thanit Saeliw, Surangrat Thongkorn, Pawinee Panjabud, Kasidit Kasitipradit, Pattanachat Lertpeerapan, Kwanjira Songsritaya, Wasana Yuwattana, Thanawin Jantheang, Depicha Jindatip, Valerie W. Hu, Takako Kikkawa, Noriko Osumi, Tewarit Sarachana

**Affiliations:** 1 Chulalongkorn Autism Research and Innovation Center of Excellence (Chula ACE), Department of Clinical Chemistry, Faculty of Allied Health Sciences, Chulalongkorn University, 154 Soi Chula 12, Rama 1 Road, Bangkok, Wangmai, Pathumwan 10330 Thailand; 2 Department of Biotechnology and Biomedicine (DTU Bioengineering), Technical University of Denmark, Kongens Lyngby, Denmark; 3 The Ph.D. Program in Clinical Biochemistry and Molecular Medicine, Department of Clinical Chemistry, Faculty of Allied Health Sciences, Chulalongkorn University, Bangkok, Thailand; 4 The M.Sc. Program in Clinical Biochemistry and Molecular Medicine, Department of Clinical Chemistry, Faculty of Allied Health Sciences, Chulalongkorn University, Bangkok, Thailand; 5 Department of Anatomy, Faculty of Medicine, Chulalongkorn University, Bangkok, Thailand; 6 Department of Biochemistry and Molecular Medicine, School of Medicine and Health Sciences, The George Washington University, Washington, DC USA; 7 Department of Developmental Neuroscience, Centers for Advanced Research and Translational Medicine (ART), Graduate School of Medicine, Tohoku University, Sendai, Miyagi Japan

**Keywords:** Sex difference, Endocrine-disrupting chemical, Bisphenol A, Autism spectrum disorder, Prefrontal cortex, Neuritogenesis, Neuronal migration, Cortical development, Social behaviors, Micro/nanoplastics

## Abstract

**Background:**

Recent studies have shown that prenatal BPA exposure altered the transcriptome profiles of autism-related genes in the offspring’s hippocampus, disrupting hippocampal neuritogenesis and causing male-specific deficits in learning. However, the sex differences in the effects of prenatal BPA exposure on the developing prefrontal cortex, which is another brain region highly implicated in autism spectrum disorder (ASD), have not been investigated.

**Methods:**

We obtained transcriptome data from RNA sequencing analysis of the prefrontal cortex of male and female rat pups prenatally exposed to BPA or control and reanalyzed. BPA-responsive genes associated with cortical development and social behaviors were selected for confirmation by qRT-PCR analysis. Neuritogenesis of primary cells from the prefrontal cortex of pups prenatally exposed to BPA or control was examined. The social behaviors of the pups were assessed using the two-trial and three-chamber tests. The male-specific impact of the downregulation of a selected BPA-responsive gene (i.e., *Sema5a*) on cortical development in vivo was interrogated using siRNA-mediated knockdown by an in utero electroporation technique.

**Results:**

Genes disrupted by prenatal BPA exposure were associated with ASD and showed sex-specific dysregulation. *Sema5a* and *Slc9a9*, which were involved in neuritogenesis and social behaviors, were downregulated only in males, while *Anxa2* and *Junb*, which were also linked to neuritogenesis and social behaviors, were suppressed only in females. Neuritogenesis was increased in males and showed a strong inverse correlation with *Sema5a* and *Slc9a9* expression levels, whereas, in the females, neuritogenesis was decreased and correlated with *Anxa2* and *Junb* levels. The siRNA-mediated knockdown of *Sema5a* in males also impaired cortical development in utero. Consistent with *Anxa2* and *Junb* downregulations, deficits in social novelty were observed only in female offspring but not in males.

**Conclusion:**

This is the first study to show that prenatal BPA exposure dysregulated the expression of ASD-related genes and functions, including cortical neuritogenesis and development and social behaviors, in a sex-dependent manner. Our findings suggest that, besides the hippocampus, BPA could also exert its adverse effects through sex-specific molecular mechanisms in the offspring’s prefrontal cortex, which in turn would lead to sex differences in ASD-related neuropathology and clinical manifestations, which deserves further investigation.

**Supplementary Information:**

The online version contains supplementary material available at 10.1186/s13293-024-00614-2.

## Highlights

Prenatal BPA exposure altered the transcriptome-interactome profiles of ASD-related genes in the offspring’s prefrontal cortex in a sex-specific pattern.

Primary prefrontal cortical neurons isolated from male pups prenatally exposed to BPA exhibited increased neuritogenesis, while that was decreased in the cells from female pups.

Deficits in social novelty were observed only in female pups prenatally exposed to BPA but not in males.

*Sema5a* and *Slc9a9* were reduced and showed an inverse correlation with neuritogenesis only in male pups exposed to BPA but not in females, suggesting that these genes may negatively regulate neuritogenesis in a male-specific manner.

*Anxa2* and *Junb* were suppressed and showed a correlation with impaired social novelty behavior only in female pups exposed to BPA but not in males, suggesting that these genes are involved in BPA-mediated social impairment in females only.

siRNA-mediated knockdown of *Sema5a* in male embryos increased neuronal migration in the developing cortex.

**Supplementary Information:**

The online version contains supplementary material available at 10.1186/s13293-024-00614-2.

## Plain English Summary

This study is the first to reveal that there was a sex difference in the effects of prenatal BPA exposure on ASD-related genes and the development of offspring’s prefrontal cortex, which is a brain region highly implicated in ASD-related behaviors. We found that prenatal BPA exposure increased neurite formation and branching of the neurons from the prefrontal cortex of male pups but suppressed such functions in females. In contrast, impaired social behaviors were observed only in female pups prenatally exposed to BPA but not in males. *Sema5a* and *Slc9a9* were reduced and showed an inverse correlation with neurite formation only in male pups but not in females, suggesting that these genes may negatively regulate neuritogenesis in a male-specific manner. On the other hand, *Anxa2* and *Junb* were suppressed and exhibited a correlation with impaired social behaviors only in female pups but not in males, suggesting that these genes are involved in BPA-mediated social impairment in females only. *Sema5a* suppression in the developing cortex of male offspring caused abnormal neuronal migration, suggesting that the male-specific downregulation of *Sema5a* caused by prenatal BPA exposure can cause abnormal development of the prefrontal cortex. In conclusion, we found that prenatal BPA exposure dysregulated cortical development and social behaviors potentially through genes and sex-specific mechanisms, which may lead to the sex difference of ASD and warrants further investigation.

## Background

Autism spectrum disorder (ASD) is an early-onset neurodevelopmental disorder that is characterized by deficits in social communication/interaction and restricted interests or repetitive patterns of behavior, according to the Diagnostic and Statistical Manual of Mental Disorders, 5th Edition (DSM-5) [1]. The United States Centers for Disease Control and Prevention’s Autism and Developmental Disabilities Monitoring (ADDM) Network has estimated the prevalence of ASD to be approximately 1 in 36 children, and males are four times more likely than girls to have ASD [2]. Although the exact causes and the underlying mechanisms of the sex bias of ASD are still unclear, both genetic and environmental factors, as well as epigenetic factors, have been associated with the etiology and the susceptibility of ASD [3–9]. One of the environmental risk factors linked to the heightened risk of ASD is exposure to endocrine-disrupting chemicals (EDCs) [3, 10, 11], which are defined as a group of chemical compounds that have the potential to disrupt the sex hormone system and impair homeostasis, reproduction, and developmental processes [3]. Examples of EDCs that have been associated with ASD susceptibility are bisphenol A (BPA) [12], nonylphenol [13], phthalates [14], and polybrominated diphenyl ethers [15].

BPA ((CH_3_)_2_C(C_6_H_4_OH)_2_) is an organic synthetic compound frequently used in the production of polycarbonate plastics and epoxy resins, which are materials that can be found in a variety of consumer products, including food and beverage containers, thermal paper receipts, dental sealants, and medical devices [3, 16–18], as well as in micro/nanoplastics which are emerging environmental pollutants in water, land, air, and groundwater environments [19]. BPA exposure can occur through ingestion, absorption through the skin, and inhalation [20–22] and can circulate throughout the body [23]. Several studies have reported that BPA can be detected in the serum and urine of pregnant women and children. BPA can readily cross the placenta [24, 25] and the blood-brain barriers [26, 27] and can be detectable in the brains of offspring whose mother is exposed to BPA during pregnancy [28].

Recent studies have revealed the association between BPA exposure and social interactions and communication in humans [29–31]. Braun et al. (2009) investigated the association between prenatal exposure to BPA and the behavior of 2-year-old children [29]. BPA concentrations were measured in maternal urine collected at different gestational stages, and child behavior was assessed using the Behavioral Assessment System for Children-2 (BASC-2). They found that maternal urinary BPA concentrations collected around 16 weeks were associated with externalizing scores among all children, and this association was stronger in females than in males [29]. Notably, BPA concentrations measured around 16 weeks of gestation showed a stronger association with externalizing scores compared to measurements at later stages [29]. In addition, Lim et al. (2017) have investigated the associations between prenatal and postnatal BPA exposure and social impairments in children [31]. They found a significant association between creatinine-adjusted prenatal urine BPA levels of second-trimester pregnant women and the Korean Social Communication Questionnaire (K-SCQ) scores of 4-year-old children born to these women [31]. They also reported that an increase in postnatal BPA exposure was significantly associated with an increase in deficits in social communication in 4-year-old girls [31]. This study provided evidence that prenatal and postnatal BPA exposure was associated with social impairment at 4 years of age, particularly in girls. Ibroci et al. (2022) have also assessed associations between prenatal maternal urinary BPA levels and the Behavior Assessment System for Children-2 (BASC-2) and Social Responsiveness Scale-2 (SRS-2) scores when their daughters were 4-5 years of age [30]. Interestingly, they found that maternal urinary BPA levels were significantly associated with daughters’ higher SRS-2 Total Scores, which indicated more pronounced social deficits, in a subgroup of the participants [30]. Other studies also investigated the associations between prenatal exposure to BPA and child behaviors and revealed the sex differences in the relationships between BPA exposure and behaviors [32–34]. Although findings about the association between prenatal BPA exposure and behaviors in boys and girls are still inconsistent, accumulating evidence strongly suggests that prenatal BPA exposure may have sex-specific effects on child behaviors. However, the molecular mechanisms underlying the sex-dependent effects of BPA on brain development and child behaviors remain unclear.

Recent studies using rodent models have shown that BPA exposure disrupted neurological functions in several brain regions, including the hypothalamus, the hippocampus, and the prefrontal cortex [35–39]. Animal studies have shown that BPA can affect brain development, leading to changes in behavior and learning ability associated with ASD [40–42]. Exposure to BPA from the embryonic period until the weaning period disrupted genes related to the insulin signaling pathway in the offspring’s hippocampus, causing impaired cognitive function in offspring [43]. Drzewiecki et al. [44] found that postnatal exposure to BPA in a short time could affect the social interaction behavior of rats in a sex-specific pattern [44].

At cellular levels, exposure to BPA caused impaired neurite outgrowth and other neuronal functions in vivo and in vitro [45–50], which are related to ASD pathophysiology [51, 52]. Our previous studies have found that prenatal exposure to BPA altered the expression of ASD-related genes in the offspring’s hippocampus, disrupting neuronal viability, neuritogenesis, synaptogenesis, and learning/memory in a sex-dependent manner [37–39]. In addition to the hippocampus, we have also demonstrated that prenatal BPA exposure had sex-specific effects on the transcriptome-interactome profiles in the offspring’s prefrontal cortex, potentially through the androgen receptor (Ar), estrogen receptor alpha (Esr1), retinoic acid-related orphan receptor-alpha (Rora), and other ASD-related transcription factors [36]. However, the sex differences in the effects of prenatal exposure to BPA on the development and functions of the offspring’s prefrontal cortex and social behaviors associated with ASD, as well as the genes underlying such sex-specific effects, are still unknown.

In this study, we, therefore, sought to examine the sex differences in the effects of prenatal exposure to BPA on the transcriptome profiles of the offspring’s prefrontal cortex, neuritogenesis of offspring’s prefrontal cortical neurons, and ASD-related social behaviors, as well as identify BPA-responsive genes associated with these effects. First, we obtained transcriptome profiling data from RNA-seq analysis of prefrontal cortex tissues isolated from male and female neonatal rat pups prenatally exposed to BPA or vehicle control. We then identified differentially expressed genes (DEGs) in response to prenatal BPA exposure in male and female pups. Gene ontology analysis of BPA-responsive genes was performed to predict DEGs significantly associated with neuritogenesis, cortical development, behaviors, or ASD. Those BPA-responsive genes were then selected for further confirmation by quantitative RT-PCR analysis. In addition, we assessed neurite formation and branching using primary cortical cells isolated from the prefrontal cortex of male and female neonatal rat pups prenatally exposed to BPA or vehicle control. The two-trial and three-chamber tests were also performed to assess the sex-specific effects of prenatal BPA exposure on the social behaviors of the male and female offspring. The correlation analyses between the expression levels of BPA-responsive genes associated with cortical neuritogenesis or social behaviors were performed to examine the relationships between those genes and the neurological functions altered by prenatal BPA exposure. As prenatal BPA exposure significantly suppressed *Sema5a* expression in the prefrontal cortex of male neonatal pups while, in the females, the expression of *Sema5a* was not affected by BPA, and additionally, an inverse correlation between *Sema5a* expression levels and neuritogenesis was observed only in the male pups but not females, we then further confirm whether such downregulation of *Sema5a* during the embryonic stage could disrupt the cortical development in male offspring. The siRNA-mediated knockdown of *Sema5a* in the developing cortex of male pups was performed using an in-utero electroporation technique, and the neuronal differentiation and migration were examined using confocal immunofluorescence analysis.

## Methods

### Collection of transcriptome profile data and the lists of DEGs in response to prenatal BPA exposure in male and female offspring

DNBseq sequencing data of total RNA extracted from prefrontal cortex tissues of BPA and vehicle control were re-analyzed by BGI Genomics Co., Ltd. Firstly, FASTQ files obtained from our previous transcriptomic data (accession no. GSE229073; https://www.ncbi.nlm.nih.gov/gds/) were quantified using SOAPnuke package (version 1.5.2) for removing adaptor, reads containing N content more than 5%, and reads with low-quality score [53]. Subsequently, clean reads in FASTQ format were aligned to the rat reference genome Rnor_6.0 (RefSeq ID: 1174938) using Hierarchical Indexing for Spliced Alignment of Transcripts (HISAT, version 2.0.4) [54]. After clean reads passed the QC of alignment, we performed gene expression quantification and differential expression analysis. Briefly, clean reads were mapped to the reference sequence of genes (transcriptome) using the Bowtie2 package (version 2.2.5) [59] and were then counted for gene expression levels using the RSEM package (version 1.2.8) [55]. The PossionDis method [56] was used for differential gene detection between BPA and vehicle control. Differential genes with P value and false discovery rate (FDR) in multiple testing less than 0.05 were considered statistically significant [56, 57].

### Prediction of neurological functions, disorders, and interactome networks associated with BPA-responsive genes

Neurological functions, disorders, and interactome networks associated with BPA-responsive genes were predicted using IPA (QIAGEN Inc., https://www.qiagenbioinformatics.com/products/ingenuity-pathway-analysis/, accessed on 20 Jan 2023) [58]. The lists of BPA-responsive genes overlapped with the list of genes experimentally validated to be associated with each function or disorder in Ingenuity’s Knowledge Base database. Fisher’s exact test was then performed to calculate P values, and a P value of < 0.05 was considered statistically significant.

### Animal husbandry and treatment

Eight-week-old male and female Wistar Furth rats were obtained from the National Laboratory Animal Center (NLAC), Thailand. All animals were housed at the Chulalongkorn University Laboratory Animal Center (CULAC) under standard temperature (21 ± 1 °C) and humidity (30–70%) conditions in a 12-h light/dark cycle with food and sterile water available *ad libitum*. Animal treatment was performed as previously described [36–38]. Briefly, female rats were mated with male rats by putting one female and one male rat per cage. Female rats were then divided into two groups which were the BPA treatment group and the vehicle control treatment group, and weighed daily to calculate the amount of BPA or ethanol vehicle control needed for treatment on each day. The female rats in the BPA treatment group were intragastrically gavaged with BPA daily by diluting 250 mg/ml of BPA in absolute ethanol (molecular biology grade) to the final concentration of 5000 µg/kg·maternal body weight from the first day after mating until parturition. This dose was equivalent to the no-observed-adverse-effect level (NOAEL) for BPA determined by the US Food and Drug Administration (FDA) and the European Food Safety Authority (EFSA) [59, 60]. For the vehicle control treatment group, absolute ethanol (molecular biology grade) in amounts equivalent to those used for preparing BPA was mixed with corn oil and fed to the rats by intragastric gavage. Rats in each condition were raised separately in individually ventilated cages. A separated set of gavage needles and all consumable products was used for each treatment condition. Reusable materials were cleaned with ethanol and rinsed with copious amounts of Milli-Q deionized water before use. All experimental procedures were approved by the Chulalongkorn University Animal Care and Use Committee (Animal Use Protocol No. 1673007, 1773011, and 2073011), Chulalongkorn University.

Rat pups (postnatal days 1-2, PND1-2) were deeply anesthetized by intraperitoneal injection of 100 mg/kg·BW sodium pentobarbital and euthanized by decapitation. The brain was quickly and carefully removed from the skull and placed in a prechilled cell culture dish containing ice-cold, freshly prepared dissection solution containing 1X HBSS (Invitrogen, USA), 30 mM glucose (Sigma-Aldrich, USA), 2 mM HEPES (GE Healthcare Bio-Sciences, USA), and 26 mM NaHCO_3_ (Sigma-Aldrich, USA). The meninges were removed entirely. Prefrontal cortex tissues were dissected under a Nikon SMZ18 Stereo Microscope (Nikon, Japan), immediately put in a tube with RNA stabilization reagent (RNAlater, Ambion, USA), and stored at -80 °C until use.

### RNA isolation

Total RNA was extracted from the prefrontal cortex of rat pups and purified using the mirVana™ miRNA Isolation Kit (Thermo Fisher Scientific, USA) according to the manufacturer’s protocol. The protocol was described in our previous study [36–38]. Briefly, prefrontal cortex tissues were lysed in a denaturing lysis buffer to stabilize RNA and inactivate RNases. Then, the lysates were subjected to acid-phenol: chloroform extraction to purify RNA and remove DNA. Ethanol was added to the samples and passed through a glass-fiber filter cartridge that immobilized the RNA. The filter was then washed three times, and finally, total RNA was eluted with a low ionic-strength solution. The purity of total RNA was determined using a NanoDrop spectrophotometer (Thermo Fisher Scientific, USA) and quantified using an Invitrogen Qubit 2.0 Fluorometer (Thermo Fisher Scientific, USA).

### Quantitative RT-PCR analysis

Male and female neonatal rat pups from five independent litters of each treatment group (*n* = 5 pups/sex/treatment group; one male and one female from each litter) were used for qRT-PCR analysis. The total RNA samples were transcribed to cDNA using a RevertAid First Strand cDNA Synthesis Kit (Thermo Scientific, USA) following the manufacturer’s protocol. Briefly, 0.5 µg total RNA was mixed with 0.2 µg random hexamer primer, and nuclease-free water was added to 12 µL. The reaction was incubated at 65 °C for 5 min and then placed on ice. After that, cDNA synthesis reagents consisting of 4 µL of 5X Reaction Buffer, 1 µL of RiboLock RNase Inhibitor (20 U/µl), 2 µL of 10 mM dNTP Mix, and 1 µL of RevertAid M-MuLV Reverse Transcriptase (200 U/µl) were added to the mixture and brought the total volume to 20 µL. Reverse transcription was performed by incubation at 25 °C for 5 min, followed by 42 °C for 60 min. The reaction was terminated by heating the solution to 70 °C for 5 min.

Quantitative PCR analysis was performed using iTaq Universal SYBR Green Supermix (Bio-Rad, USA) according to the manufacturer’s instructions. Briefly, 1 µl of cDNA was mixed with 2X iTaq Universal SYBR Green Supermix, 500 nM forward primer, 500 nM reverse primer, and nuclease-free water to the final volume of 10 µl. Triplicate reactions were prepared for each sample. The total reaction mixture was then incubated in a Bio-Rad CFX Connect Real-Time PCR Detection System (Bio-Rad, USA). The PCR conditions were set as follows: the polymerase activation step at 95 °C for 30 s, the initial denaturing step at 95 °C for 15 min, and followed by 40 cycles of 95 °C for 10 s per cycle and 30 s at 55 °C for annealing/extension. Melting curve analysis was set at 65 to 95 °C for product confirmation. The expression levels were calculated by the 2^−ΔΔCt^ method using the 18S ribosomal RNA (*Rn18s*) gene as an endogenous control. The primers used in this study were designed using the UCSC Genome Browser (https://genome.ucsc.edu/) [61], Ensembl (https://asia.ensembl.org/index.html) [62], and Primer3 software (http://bioinfo.ut.ee/primer3-0.4.0/) [63–65]. Forward and reverse primers were designed for rat genes, including *Anxa2*, *Junb*, *Nrp2*, *Slc9a9*, *Sema5a*, *P2rx4*, *Arhgap32*, *Aif1*, and *Rn18s*. The sequences of all primers are shown in Additional file 1.

### Neurite formation assay

To investigate the sex difference in the effect of prenatal BPA exposure on neurite formation, we isolated primary prefrontal cortical neural cells from the prefrontal cortex of neonatal rat pups PND1-2 from the BPA and the control treatment group as described previously with slight modification [66, 67]. Briefly, the prefrontal cortex tissues were dissected under a stereomicroscope (Nikon, Japan) in the dissection medium. Then, primary cortical cells were isolated from the tissues using 0.25% trypsin (Gibco™, Thermo Fisher Scientific, USA) and incubated at 37 ^o^C for 20 min. Next, 10 mg/ml DNase solution (Sigma-Aldrich, USA) was added to the tubes and incubated at room temperature for 5 min. The tissue suspension was washed by dissection solution and then mixed with 2.5 mL of plating medium containing 86.55% MEM Eagle’s with Earle’s BSS, 10% fetal bovine serum (Sigma-Aldrich, USA), 1X 100 mM Sodium pyruvate (Gibco™, Thermo Fisher Scientific, USA), 0.45% of 100 g/L glucose solution (Sigma-Aldrich, USA), 1X of 200 mM L-glutamine (Gibco™, Thermo Fisher Scientific, USA), and 1X of 100X penicillin/streptomycin (Gibco™, Thermo Fisher Scientific, USA). The tissues were then triturated using a fire-polished glass Pasteur pipette. After that, the cells were counted and seeded on a 22 × 22 mm poly-L-lysine coated coverslip placed in a 35 mm cultured dish (Falcon, USA) at a density of 1 × 10^4^ cells/cm^2^. The cells were incubated in a 5% CO_2_ and 37 ^o^C incubator for 4 h to allow the cells to attach a coverslip. Then, 3 mL of maintenance medium containing 96% Neurobasal medium (Gibco™, Thermo Fisher Scientific, USA), 1X of 50X B-27 (Gibco™, Thermo Fisher Scientific, USA), 1X of 100X 200 mM glutamine (Gibco™, Thermo Fisher Scientific, USA), 1X of 100X penicillin/streptomycin (Gibco™, Thermo Fisher Scientific, USA) was added to the dish until the medium covered all areas of the culture dish and then incubated in 5% CO_2_ and 37 ^o^C incubator. The maintenance medium was changed in half volume every two days.

The morphological changes of neurons were observed under a bright-field microscope. To quantify the neurite formation of primary cortical cells, neurons (*n* = 80-100 cells/sex/treatment group) were imaged after cultured for 14 days. The parameters used in this analysis were the number of branches of neurite, total neurite length, primary neurite length, average branch length, number of primary neurites/neuron, and number of branches/neuron. In addition, Sholl analysis, which is a widely used method in neurobiology to determine dendritic arborization complexity, was also performed by plotting the number of dendrite intersections against the radial distance from the soma center by using ImageJ software [68–72].

### The two-trial social interaction test

The two-trial social interaction test was used to assess the ability of rat offspring to recognize novel versus familiar rats and assess two-way social communication. This test was performed in an open-field square arena as previously described (https://med.stanford.edu/sbfnl/services/bm/si/two-trial.html). The subject rats in the BPA treatment group (*n* = 20; 10 males and 10 females) and vehicle control group (*n* = 19; 9 males and 10 females) aged 33-34 days and the two novel stimulus rats (age- and gender-matched rats) were habituated to the testing room for 1 h before starting the test. Initially, the subject rat was placed in the center of the open field and habituated the arena for 15 min. Another novel rat was then placed in the arena. Both rats were freely allowed to spend time interacting for 5 min. After that, the novel rat was brought back to the home cage for 5 min while the subject rat was still in the arena alone. Next, the met-before and new rats were placed in the arena together. Those rats were left in the arena for 5 min and taken back to their home cage. All test trials were videotaped and subsequently analyzed for the time that the subject rat used in each parameter. The parameters used in this experiment were (1) the time that the subject rat used its nose to sniff the nose or the genital area of the met-before rat (i.e., familiar rat) or the new rat (i.e., novel rat), (2) the total interacting time that the subject rat spent with the familiar rat and novel rat, and (3) the preference index of two-trial, which was the difference of time spent interacting with a first and second rat over the total time spent interacting with both rats.

### The three-chamber test

Rat pups aged 43-44 days (the BPA treatment group: *n* = 12 pups, 6 males and 6 females; the vehicle control group: *n* = 10 pups, 6 males and 4 females) were used. The three-chamber test was performed as previously described [73] with slight modification. The apparatus utilized in the experiment was a chamber divided into three parts. Two small cages were placed in the center of the left and right sides of the chamber. The test consisted of three sessions with no inter-session intervals. In the first session, a subject rat was placed in the center chamber and habituated to the three chambers for 5 min. The subject rat was then removed, and the chamber was thoroughly cleaned with 70% ethanol to clear any scent, urine, or feces. The subject rat was then placed back in the center chamber while another rat (age- and gender-matched), called a stranger rat, was randomly placed in a small cage on one side while the other chamber was left empty. The subject rat was allowed to explore the entire chamber freely for 10 min. The sociability was determined by measuring the time that the subject rat used in 3 parameters: the time that the subject rat spent interacting with (1) the stranger rat or (2) an empty chamber, (3) the preference index of sociability was also calculated by determining the ratio between the difference in time spent in the chamber containing the stranger rat versus the empty chamber, divided by the sum of the time spent in both chambers. In the next session, another rat as novel rat (age- and gender-matched) was placed in the previously emptied chamber. Then, the subject rat was allowed to explore the whole chamber freely for 10 min. The social novelty parameters consist of 8 parameters: the time that the subject rat spent interacting with (1) the familiar or (2) the novel rat, (3) the total time that the subject rat spent interacting with both the familiar and novel rat, (4) the preference index of the time spent interacting was also determined by calculating the ratio between the difference in time spent interacting with the familiar and novel rat over the sum of the time spent interacting with both rats, the time that the subject rat spent in the chamber containing (5) the familiar or (6) the novel rat, (7) the total time that the rat spent in both familiar and novel rat, and (8) the preference index of the time that the subject rat spent in the chamber was also determined by calculating the ratio between the difference in time spent in the chamber containing the familiar and novel rat over the sum of the time spent in the chamber containing both rats.

### Correlation analysis

Correlation analysis was performed to examine the possibility of the association between the gene expression level in response to BPA and the changes in neurological function. To perform an association between BPA-responsive genes and behavior parameters, we conducted Pearson’s correlation analysis as described in the Pavlidis Template Matching (PTM) method [74]. Briefly, each behavior parameter was adjusted to 0 to 1 values and used as the template match to the expression level of each gene from RNA-sequencing data (normalized read counts). A P value less than 0.05 was a threshold for a significant template match. Moreover, expression values of selected DEGs validated by qRT-PCR analysis and the changes in neurological function were used to perform correlation analysis by matching each log_2_(fold change) expression value with a behavior phenotype from the behavioral tests or the parameters from the neurite formation experiment. Pearson’s correlation coefficient was used to calculate the correlation coefficient (R values). The R values, ranging from − 1 to + 1, were then used to construct a heatmap in both sexes, males and females. A positive value indicates a positive correlation, while a negative indicates an inverse correlation.

### In situ hybridization

To create the *Sema5a* insert sequence, we extracted total RNAs from E14.5 wild type (C57BL/6J) mouse brain embryos using the RNeasy Mini Kit (Qiagen, Germany) according to the manufacturer’s protocol and reverse transcribed to complementary DNA (cDNA) using the Invitrogen™ SuperScipt III™ First-Strand Synthesis Supermix (Thermo-Fisher Scientific, USA) according to the manufacturer’s protocol. The cDNAs were used as a template for amplifying the *Sema5a* insert sequence using primers as follows: *Sema5a* Forward 5’- CGGTATCGATAAGCTAGCTGGTCAGAGTGGTCA-3’ and *Sema5a* Reverse 5’- CGGGCTGCAGGAATTGGAATAGGTCTTCCCAGTG-3’. Amplification was performed with a thermal cycler (Mastercycler Gradient; Eppendorf, Germany) using PrimeSTAR HS DNA Polymerase (Takara Bioscience, Japan) with the following protocol: polymerase activation at 98 °C for 2 min, denaturation at 98 °C for 10 s, annealing at 50.8 °C for 5 s, extension for 1 min at 72 °C, 30 cycles. The cDNA fragments were then purified using NucleoSpin Gel and PCR Clean-up (Macherey-Nagel, Germany) according to the manufacturer’s protocol. The purified cDNA fragments were cloned into pBluescript II SK (-) (pBSK(-); Stratagene, USA). Plasmid DNA sequencing was used to confirm whether the *Sema5a* sequence was inserted into the plasmid. The pBSK(-) that contained *Sema5a* insert (pBSK(-)-*Sema5a*) was linearized using a restriction enzyme. The antisense riboprobes were synthesized using T3 RNA polymerase (Promega, USA) with linearized pBSK(-)-*Sema5a* as the template and labeled with Digoxigenin-11-UTP (DIG; Roche, Switzerland).

The in situ hybridization was performed as described previously [75–78]. Briefly, the 4% paraformaldehyde-fixed frozen coronal sections of the E14.5 embryonic brain were treated with Proteinase K (Roche, Switzerland) and incubated in the hybridization buffer containing *Sema5a* antisense riboprobes at 70 ^o^C for 14 h. Then, the sections were washed and blocked with 0.5% Roche blocking reagent (Roche, Switzerland) for 1 h at room temperature for nucleic acid hybridization and detection and then incubated with 1:4000 alkaline phosphatase-conjugated anti-Digoxigenin, Fab fragments (Roche, Switzerland) at 4 ^o^C overnight. The signals were observed with its color-indicating substrate, nitro-blue tetrazolium chloride (NBT) and 5-bromo-4-chloro-3’-indolyphosphate p-toluidine salt (BCIP) that could yield an intense, insoluble black-purple precipitate when reacted with alkaline phosphatase. The color reaction was stopped by washing with 1X TBST and then 1X PBS. The in situ hybridization sections were covered with a coverslip containing VECTASHIELD Antifade Mounting Medium (Vectorlabs, USA).

### In utero electroporation of the embryonic mouse brain

The in utero electroporation of *Sema5a* siRNA into the embryonic mouse brain was performed as described previously using C57BL/6J strain mice with slight modifications [79]. Animal experiments were conducted following the guidelines the National Institutes of Health set forth as outlined in the Guide for the Care and Use of Laboratory Animals. The experimental procedures described in this experiment were approved by the Committee for Animal Experimentation at Tohoku University Graduate School of Medicine. The midday of the day when the vaginal plug was observed was designated as the embryonic day 0.5 (E0.5). C57BL/6J strain mice at E14.5 were anesthetized with isoflurane using the Univentor 400 anesthesia unit (Univentor, Malta). For microinjection, 75-mm glass capillary tubes were pulled using a micropipette puller NARISHIGE PB-7 (Narishige, Japan). The injection solutions consisted of the expression vectors *pCAG-EGFP* plasmid (kindly gifted from Prof. Tetsuichiro Saito, Chiba University, Japan) at a final concentration of 0.5 µg/µL with *Sema5a* siRNA (Thermo-Fisher Scientific, USA) that had the sense strand sequence as follows: 5’-GAGAUGACGUGUGGAACCAUUUCAA-3’, and antisense strand sequence as follows: 5’-UUGAAAUGGUUCCACACGUCAUCUC-3’ or control (stealth) siRNA that had the sense strand sequences: 5’- CAGUAACAUCUCGCCAUCCCACUCA-3’, and antisense strand sequences: 5’- UGAGUGGGAUGGCGAGAUGUUACUG-3’ at a final concentration of 2 µg/µL and Fast green FCF (Nacalai Tesque, Japan) in PBS were filled in the micropipette using a syringe. The injection solutions were injected into the lateral ventricle of the embryos using Narishige microinjector IM 300 (Narishige, Japan). The forceps-type round platinum electrodes (LF650P5, BEX) were placed on the brain of embryos. The electric pulses were delivered by applying the voltage at 40 V with an interval cycle length (Pon) of 50 msec and interval pause (Poff) of 950 msec through the uterus using a CUY21 electroporator (BEX, Japan). The uterus was kept wet all the time by dropping Tyrode’s solution prewarmed at 37 °C. The embryonic brains were collected at E16.5 and E18.5 for further analysis.

### Immunofluorescence staining

The E16.5 or E18.5 electroporated mouse brains were placed in a solution of 1% paraformaldehyde in PBS, which was freshly prepared. After being kept in the solution overnight at 4 °C, the paraformaldehyde was replaced with a 10% sucrose solution in PBS, followed by another overnight soak at 4 °C in a 20% sucrose solution. The tissue was embedded in an OCT compound (Sakura Finetek, USA) and frozen. The brain was sliced into coronal sections using a Leica cryostat (Leica, USA). The sections were dried for 15 min and washed three times with TBST (0.1% Triton X-100) for 5 min. The sections were then blocked with 3% BSA for 1 h at room temperature. Secondary antibodies were added, and the sections were counterstained with DAPI, followed by incubation at room temperature for 1 h. The sections were mounted using VECTASHIELD Antifade Mounting Medium (Vectorlabs, USA). The primary antibodies were used as follows: chicken anti-GFP (1:1000; Abcam), rabbit anti-Pax6 (1:1000, PD022, MBL International Corporation), rabbit anti-Tbr2 (1:1000, 14-4875-82, Invitrogen). Secondary antibodies were used as follows: Cy3-conjugated donkey anti-rabbit IgG (1: 500; Jackson Immunoresearch Laboratories) and Alexa 488-conjugated affinity-purified anti-chicken IgY goat antibodies (1: 500; Invitrogen). 4’,6-Diamidino-2-phenylindole dihydrochloride (DAPI) (1:1000; Sigma) was used as nuclear counterstaining. GFP^+^ cells, Pax6^+^ cells, and Tbr2^+^ cells were detected using an LSM800 confocal laser scanning microscope (Carl Zeiss, Germany). The cortical layers of E16.5 sections were classified as ventricular/subventricular zone (VZ/SVZ), intermediate zone (IZ), and cortical plate (CP). In addition, the cortical layers of E18.5 sections were divided into 10 equal layers (10 bins). The numbers of GFP^+^ cells, GFP^+^Pax6^+^ cells, and GFP^+^Tbr2^+^ cells were counted using ImageJ software [68].

### Statistical analyses

Two-way ANOVA, followed by Tukey’s Honest Significant Difference (HSD) posttest, was employed to compare gender effects and the effects of treatment in the qRT-PCR, two-trial and three-chamber social behavior tests, and neurite formation assay. The percentages of GFP^+^ cells, GFP^+^Pax6^+^ cells, and GFP^+^Tbr2^+^ cells were used to compare the mean values between the *Sema5a* siRNA group and the control siRNA group in each layer/bin using a One-way ANOVA, followed by Tukey HSD’s multiple comparison tests. A P value < 0.05 was considered statistically significant.

## Results

### Sex-specific effects of prenatal BPA exposure on the transcriptome-interactome profiles of genes associated with neuritogenesis, behaviors, and ASD in the offspring’s prefrontal cortex

Our previous study has shown that prenatal BPA exposure altered the transcriptome-interactome profiles of the prefrontal cortex of neonatal rats [36]. Moreover, the list of BPA-responsive genes was significantly enriched with known ASD candidate genes, as well as genes that were dysregulated in the postmortem brain tissues of ASD cases from multiple independent studies [36]. However, the sex differences in the effects of prenatal BPA exposure on neurological functions in the offspring’s prefrontal cortex and related behaviors are still unknown. To identify neurological functions and behaviors influenced by prenatal BPA exposure for further investigations, we reanalyzed our previously published data from transcriptome profiling analysis of prefrontal cortex tissues isolated from male and female neonatal rat pups prenatally exposed to BPA or ethanol as vehicle control using a modified bioinformatic pipeline as described in the Methods section. These data are available in the NCBI Gene Expression Omnibus (GEO) DataSets database (accession: GSE229073; https://www.ncbi.nlm.nih.gov/gds/).

Regarding the samples used for this transcriptome profiling analysis, RNA samples were isolated from prefrontal cortex tissues of neonatal rat pups (postnatal day 1, PND1) whose mothers were exposed to BPA daily at a dose of 5000 µg/kg maternal body weight/day (BPA *n* = 6, consisting of 3 male and 3 female pups) or vehicle control (control *n* = 6, consisting of 3 male and 3 female pups) from gestational day 0 (GD0) until parturition. All replicates were obtained from independent litters. When considering both male and female pups collectively, a total of 591 genes were found to be significantly differentially expressed in the prefrontal cortex of pups in response to prenatal exposure to BPA compared to the vehicle control group (Additional file 2). When the transcriptome profiles of male and female pups were analyzed separately, a total of 4488 and 4658 genes were found to be differentially expressed in response to BPA in male and female prefrontal cortex tissues, respectively (Additional file 3, 4). Among these, as many as 1986 genes were differentially expressed only in males, whereas 2156 genes were altered only in females (Additional file 5). This finding indicates that prenatal BPA exposure causes sex-specific dysregulation of transcriptome profiles in the offspring’s prefrontal cortex.

The lists of BPA-responsive genes were then analyzed using QIAGEN IPA software (QIAGEN Inc., https://digitalinsights.qiagen.com/IPA) [58] to predict neurological functions, disorders, and interactome networks associated with the BPA-responsive genes in the prefrontal cortex. The IPA analysis unveiled a significant association between the BPA-responsive genes and neurological diseases, including “autism spectrum disorder,” “cognitive impairment,” and “movement disorder.” Additionally, it highlighted biological functions associated with cortical development, including “neuritogenesis,” and “morphology of cerebral cortex.” Furthermore, behaviors linked to BPA-responsive genes in the prefrontal cortex, including “cognition,” and “learning,” were also identified (P value < 0.05, Table 1). The interactome networks of DEGs in both sexes, male, and female pups prenatally exposed to BPA also showed the relationships between BPA-responsive genes and neurological functions/disorders, including “autism spectrum disorder,” “neurodevelopmental disorder,” “developmental delay,” “learning,” “formation of the forebrain,” and “neuritogenesis” (Additional File 6, 7, 8). These findings suggest that prenatal exposure to BPA alters the expression profiles of genes associated with ASD and related neurological functions, including neuritogenesis, cortical development, and behaviors in the prefrontal cortex of male and female offspring in a sex-specific pattern.

**Table 1:** **Neurological functions and disorders associated with DEGs in the offspring’s prefrontal cortex in response to prenatal BPA exposure.** The table lists differentially expressed genes in the prefrontal cortex of rat pups exposed prenatally to BPA. The analysis was conducted under two conditions: i) When both male and female pups were combined into one group for each treatment. ii) When each sex of pups was analyzed separately using IPA software. The predicted neurological functions and disorders associated with these BPA-responsive genes were determined. Statistical significance was assessed using Fisher’s exact test, and the resulting P values were reported. A P value < 0.05 is considered as significant

Categories	P value	# DEGs
***Both sexes***		
*Neurological diseases and function*		
- Movement disorders	5.99E-27	137
- Cognitive impairment	3.81E-20	99
- Autism spectrum disorder or intellectual disability	1.79E-14	81
- Neurodevelopmental disorder	4.34E-14	61
- Abnormal morphology of neurons	2.70E-13	59
*Nervous system development and function*		
- Morphogenesis of neurons	1.46E-18	86
- Neuritogenesis	8.09E-17	82
- Development of cerebral cortex	4.60E-12	29
- Morphology of forebrain	8.94E-12	33
- Morphology of cerebral cortex	1.30E-09	29
*Behaviors*		
- Learning	1.11E-10	48
- Cognition	1.14E-10	51
- Memory	2.71E-09	34
- Spatial learning	1.18E-07	23
- Locomotion	2.29E-07	34
***Male***		
*Neurological diseases and function*		
- Cognitive impairment	4.79E-74	551
- Movement disorders	7.84E-67	691
- Autism spectrum disorder or intellectual disability	2.09E-64	488
- Mental retardation	1.04E-53	398
- Neurodevelopmental disorder	1.13E-46	325
*Nervous system development and function*		
- Morphogenesis of neurons	1.92E-79	497
- Neuritogenesis	5.60E-78	490
- Branching of neurites	9.24E-40	238
- Development of cerebral cortex	1.47E-27	120
- Morphology of cerebral cortex	7.78E-26	137
*Behaviors*		
- Cognition	2.58E-29	265
- Learning	1.04E-28	245
- Locomotion	1.08E-23	188
- Emotional behavior	1.52E-19	156
- Spatial learning	3.60E-18	106
***Female***		
*Neurological diseases and function*		
- Movement disorders	1.65E-72	726
- Cognitive impairment	1.99E-65	547
- Autism spectrum disorder or intellectual disability	8.14E-47	460
- Abnormal morphology of neurons	4.16E-44	325
- Epilepsy or neurodevelopmental disorder	3.34E-43	447
*Nervous system development and function*		
- Morphogenesis of neurons	2.10E-93	536
- Neuritogenesis	5.42E-92	529
- Branching of neurites	3.32E-36	237
- Morphology of cerebral cortex	1.81E-21	132
- Morphology of forebrain	4.84E-21	135
*Behaviors*		
- Cognition	2.37E-46	311
- Learning	3.05E-42	282
- Memory	1.10E-27	178
- Locomotion	1.20E-26	200
- Spatial learning	2.55E-18	109

### Quantitative RT-PCR analysis of BPA-responsive genes associated with neuritogenesis and social behaviors

**Fig. 1:**
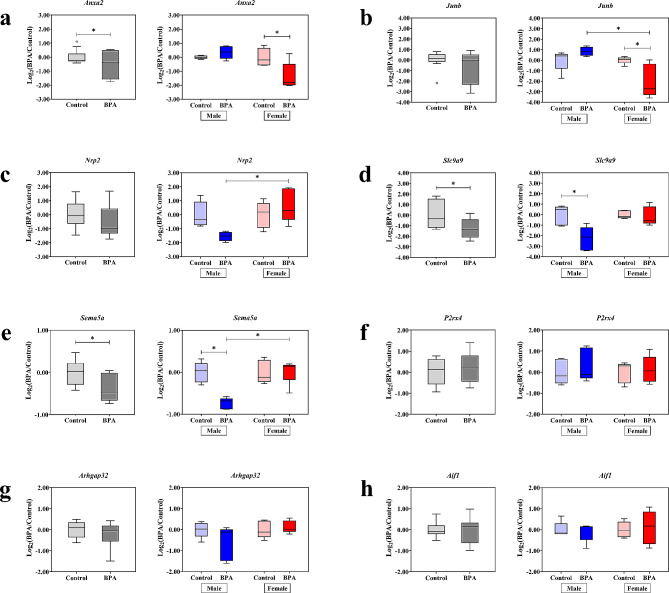
**Box plot graph of the expression levels of neurological functions, including neuritogenesis and behaviors related genes in the prefrontal cortex of offspring.** Quantitative RT-PCR analysis of the prefrontal cortex of offspring (PND0-1) prenatally exposed to BPA or the vehicle control was performed to assess the expression levels of (**a**) *Anxa2* (treatment *F*_1,16_ = 6.810 and P value = 0.019, sex *F*_1,34_ = 0.142 and P value = 0.711, P value = 0.012 for treatment × sex, by two-way ANOVA), (**b**) *Junb* (treatment *F*_1,16_ = 1.823 and P value = 0.196, sex *F*_1,16_ = 1.378 and P value = 0.258, P value = 0.005 for treatment × sex, by two-way ANOVA), (**c**) *Nrp2* (treatment *F*_1,16_ = 1.175 and P value = 0.294, sex *F*_1,16_ = 2.313 and P value = 0.148, P value = 0.014 for treatment × sex, by two-way ANOVA), (**d**) *Slc9a9* (treatment *F*_1,16_ = 9.486 and P value = 0.007, sex *F*_1,16_ = 5.355 and P value = 0.034, P value = 0.015 for treatment × sex, by two-way ANOVA), (**e**) *Sema5a* (treatment *F*_1,16_ = 10.910 and P value = 0.004, sex *F*_1,16_ = 0.547 and P value = 0.470, P value = 0.003 for treatment × sex, by two-way ANOVA), (**f**) *P2rx4* (treatment *F*_1,16_ = 0.632 and P value = 0.438, sex *F*_1,16_ = 3.829 and P value = 0.068, P value = 0.725 for treatment × sex, by two-way ANOVA), (**g**) *Arhgap32* (treatment *F*_1,16_ = 1.140 and P value = 0.301, sex *F*_1,16_ = 0.461 and P value = 0.507, P value = 0.122 for treatment × sex, by two-way ANOVA), and (**h**) *Aif1* (treatment *F*_1,16_ = 0.000 and P value = 0.995, sex *F*_1,16_ = 0.183 and P value = 0.675, P value = 0.663 for treatment × sex, by two-way ANOVA) when both sexes (grey) of offspring were combined, in male or female offspring only. *P value < 0.05

To further investigate the sex differences in the effects of prenatal exposure to BPA on transcriptome profiles of genes in the offspring’s prefrontal cortex, we selected eight BPA-responsive genes for qRT-PCR analysis (Fig. 1). Among these, three genes (i.e., *Junb*, *Nrp2*, and *Sema5a*) were associated with neuritogenesis [80–85], whereas the others (i.e., *Anxa2*, *Slc9a9*, *Arhgap32*, *Aif1*, and *P2rx4*) were associated with social behaviors [86–93]. Animal treatment and isolation of prefrontal cortex tissues were performed as described in the Methods section and previous study [36]. RNA samples were extracted from the prefrontal cortex tissues of neonatal rat pups prenatally exposed to BPA at a daily dose of 5000 µg/kg maternal body weight (*n* = 10 pups; 5 males and 5 females) or vehicle control (*n* = 10 pups; 5 males and 5 females) from GD0 until parturition. All replicates were obtained from independent litters. The results of the qRT-PCR analysis revealed that when considering both male and female pups together, the expression levels of *Anxa2*, *Slc9a9*, and *Sema5a* genes were significantly downregulated in the BPA-treated group compared to the control group (Fig. 1a, d and e). Moreover, when analyzing the expression of these genes separately in males and females, sex-specific dysregulation patterns of these genes were observed. The expression levels of *Slc9a9* and *Sema5a* were significantly reduced in response to prenatal exposure to BPA in male pups but not in females (Fig. 1d and e). In contrast, the expression levels of *Anxa2* and *Junb* were significantly decreased in females only (Fig. 1a and b).

### Sex differences in the effects of prenatal BPA exposure on the neuritogenesis of primary cortical cells in the offspring’s prefrontal cortex

**Fig. 2:**
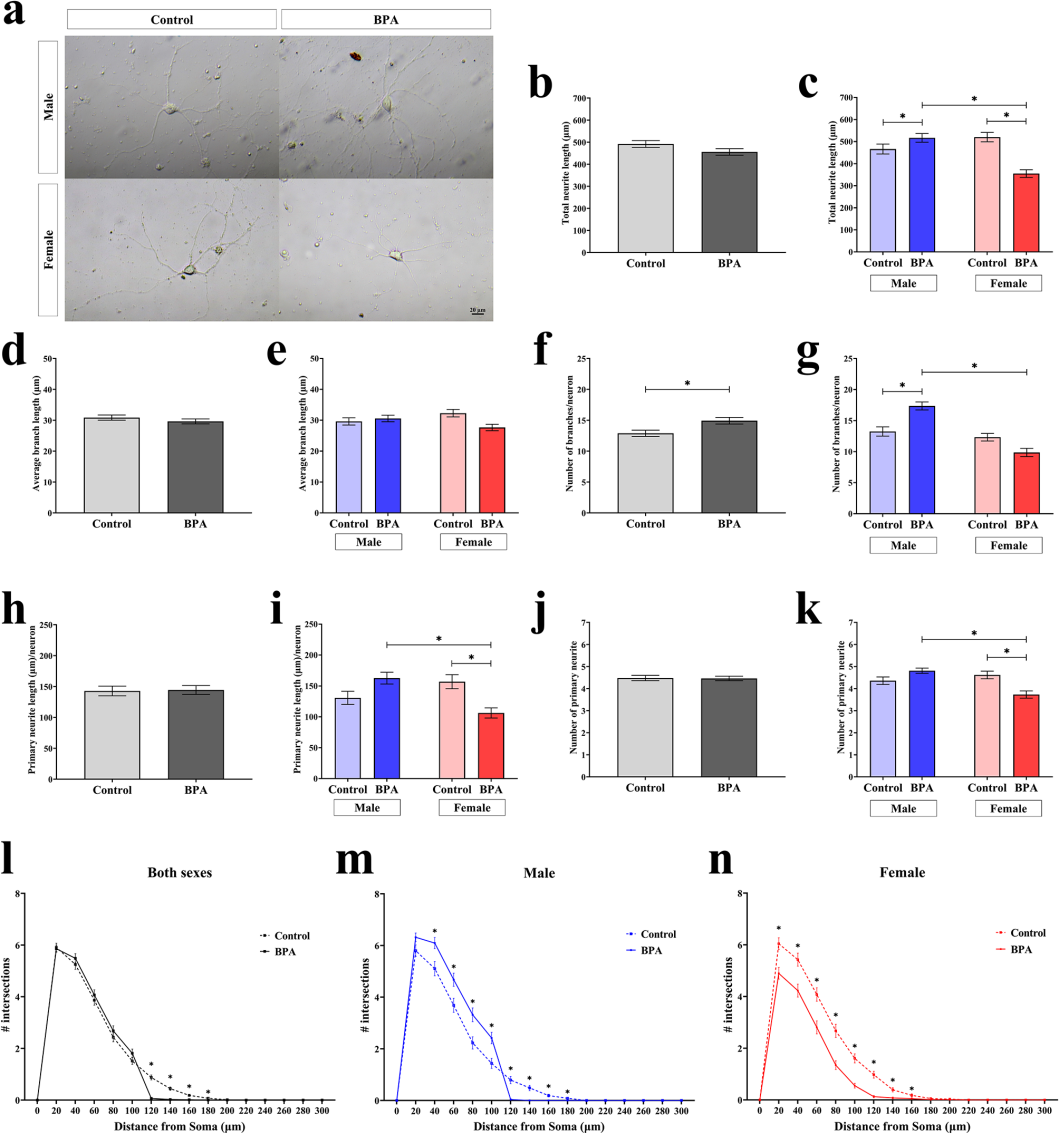
**Quantification of neurite formation in primary cortical cells isolated from the prefrontal cortex of the rat offspring prenatally exposed to BPA or vehicle control.** (**a**) Cortical cells were isolated from neonatal rat pups prenatally exposed to BPA or the vehicle control and cultured for 14 days. Images of the cells were taken using an inverted microscope. (**b, c**) Total neurite length (treatment *F*_1,34_ = 0.097 and P value = 0.756, sex *F*_1,34_ = 24.639 and P value < 0.001, P value < 0.001 for treatment × sex, by two-way ANOVA), (**d, e**) average branch length (treatment *F*_1,34_ = 2.325 and P value = 0.128, sex *F*_1,34_ = 0.011 and P value = 0.918, P value = 0.021 for treatment × sex, by two-way ANOVA), (**f, g**) number of branches per neuron (treatment *F*_1,34_ = 1.070 and P value = 0.302, sex *F*_1,34_ = 33.772 and P value < 0.001, P value < 0.001 for treatment × sex, by two-way ANOVA), (**h, i**) primary neurite length (treatment *F*_1,34_ = 1.889 and P value = 0.170, sex *F*_1,34_ = 6.667 and P value = 0.010, P value < 0.001 for treatment × sex, by two-way ANOVA), (**j, k**) number of primary neurites per neuron (treatment *F*_1,34_ = 0.744 and P value = 0.389, sex *F*_1,34_ = 1.951 and P value = 0.163, P value < 0.001 for treatment × sex, by two-way ANOVA), and (**l, m, n**) Sholl analysis of primary cortical neurite (treatment *F*_1,34_ = 14.303 and P value < 0.001, sex *F*_1,34_ = 38.658 and P value < 0.001, radius *F*_1,34_ = 780.439 and P value < 0.001, P value < 0.001 for treatment × sex × radius, by three-way ANOVA) were determined. Data are presented as the mean ± SEM. *P value < 0.05

Our previous study has shown that prenatal BPA exposure disrupts transcriptome profiles, including ASD-related genes of the offspring’s prefrontal cortex [36]. Gene ontology analysis predicted that those BPA-responsive genes were associated with several ASD-related neurological signaling pathways/functions, including axon guidance signaling, neuritogenesis, or outgrowth of neurites, in a sex-dependent manner [36]. However, the sex differences in the effects of prenatal BPA exposure on neuritogenesis of the prefrontal cortical neurons of the offspring have not been studied. To determine whether prenatal BPA exposure disrupted the neuritogenesis of offspring’s prefrontal cortical neurons in a sex-specific fashion, primary prefrontal cortical neurons were isolated from the brain of PND1 pups exposed to BPA or vehicle control during the gestation and cultured for 14 days in vitro. The prefrontal cortical cells (*n* = 80–100 cells/sex/treatment group) were then examined under a bright-field microscope (Fig. 2a). A total of six parameters including total neurite length (Fig. 2b and c), average branch length (Fig. 2d and e), number of branches per neuron (Fig. 2f and g), primary neurite length (Fig. 2h and i), number of primary neurites per neuron (Fig. 2j and k), and the number of intersections over the distance from the soma (Fig. 2l, m and n). The results showed that the number of branches per neuron was increased in the BPA treatment group (Fig. 2f). In contrast, total neurite length, primary neurite length, average branch length, and the number of primary neurites per neuron were not affected by prenatal BPA exposure. In addition, the number of intersections over the distance of neurites from neonatal rats prenatally exposed to BPA decreased at 120, 140, 160, and 180 μm (Fig. 2l). Interestingly, we found sex differences in total neurite length (Fig. 2c), the number of branches per neuron (Fig. 2g), primary neurite length (Fig. 2i), and the number of primary neurites per neuron (Fig. 2k) were decreased in the female group but increased in the male group in response to prenatal BPA exposure. Furthermore, the number of intersections over the distance of neurites from prenatal BPA exposure neonatal male offspring was increased at a distance of 40, 60, 80, and 100 μm, and increased at a distance of 120, 140, 160, and 180 μm (Fig. 2m). However, in females, the number of intersections over the distance of neurites was reduced at all distances (Fig. 2n). These results suggest that BPA exposure during the gestation period altered the neurite formation of cortical cells in the prefrontal cortex of offspring in a sex-dependent manner.

### Sex differences in the effects of prenatal BPA exposure on offspring’s social behaviors

**Fig. 3:**
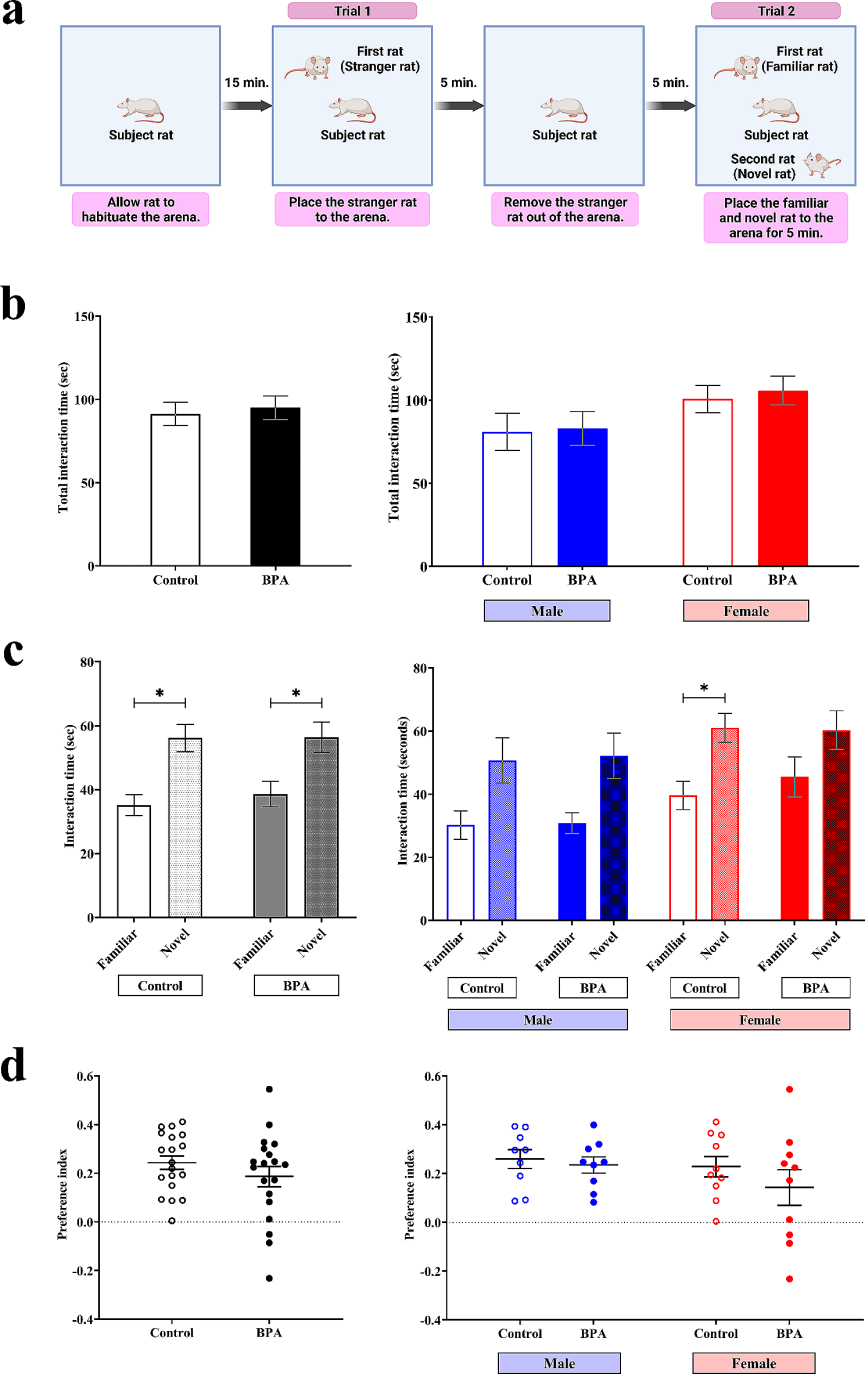
Prenatal exposure to BPA reduces social reciprocity interaction evaluated by the two-trial test in a sex-dependent manner. (**a**) The social reciprocity interaction in the offspring was assessed using two-trial tests. (**b**) The total time that the rat spent interacting with a familiar and a novel rat was measured (treatment *F*_1,34_ = 0.141 and P value = 0.709, sex *F*_1,34_ = 4.935 and P value = 0.033, P value = 0.875 for treatment × sex, by two-way ANOVA). (**c**) The times that the rat spent interacting with a familiar or a novel rat were measured (treatment *F*_1,68_ = 0.204 and P value = 0.653, sex *F*_1,68_ = 7.127 and P value = 0.009, intruder *F*_1,68_ = 24.034 and P value < 0.001 and P value = 0.640 for treatment × sex × intruder, by three-way ANOVA) and (**d**) used for calculating the discrimination index (treatment *F*_1,34_ = 1.191 and P value = 0.283, sex *F*_1,34_ = 1.493 and P value = 0.230, P value = 0.550 for treatment × sex, by two-way ANOVA). Data are presented as the mean ± SEM. *P value < 0.05

ASD cases are known to have deficits in social behaviors, including social memory, sociability, and social novelty interest [94, 95], all of which are controlled by the prefrontal cortex [96, 97]. BPA exposure has been associated with altered social behaviors in humans and animal models [31, 98, 99], but the sex differences in the effects of prenatal BPA exposure have not been investigated. To interrogate the differential effects of prenatal BPA exposure on social behaviors of male- and female offspring, we performed two social behavior tests, the two-trial and the three-chamber social tests, using rat offspring that were PND 33–34 and PND 43–49, respectively. For the two-trial test, which was used to determine whether the subject offspring make social reciprocity interaction with the familiar rat and the never-before-met rat, a total of 39 rat pups prenatally exposed to BPA (*n* = 20, 10 males and 10 females) or vehicle control (*n* = 19, 9 males and 10 females) were used (Fig. 3). All animal replicates were obtained by random selection from independent litters. The time that the subject offspring spent interacting with the familiar or novel rats was measured. The schematic diagram of this test is shown in Fig. 3a. We found that when both sexes of rat pups that received the same treatment were combined into one group, pups in both the BPA group and the vehicle control groups spent more time interacting with novel rats than with familiar rats (Fig. 3c, left), which is a common rat social behavior that could be expected. However, when each sex of pups was analyzed separately, the time that female rat pups prenatally exposed to BPA spent interacting with the familiar and the novel rats was not significantly different, unlike the control group (Fig. 3c, right), suggesting that prenatal BPA exposure impaired social novelty interest of female offspring.

**Fig. 4:**
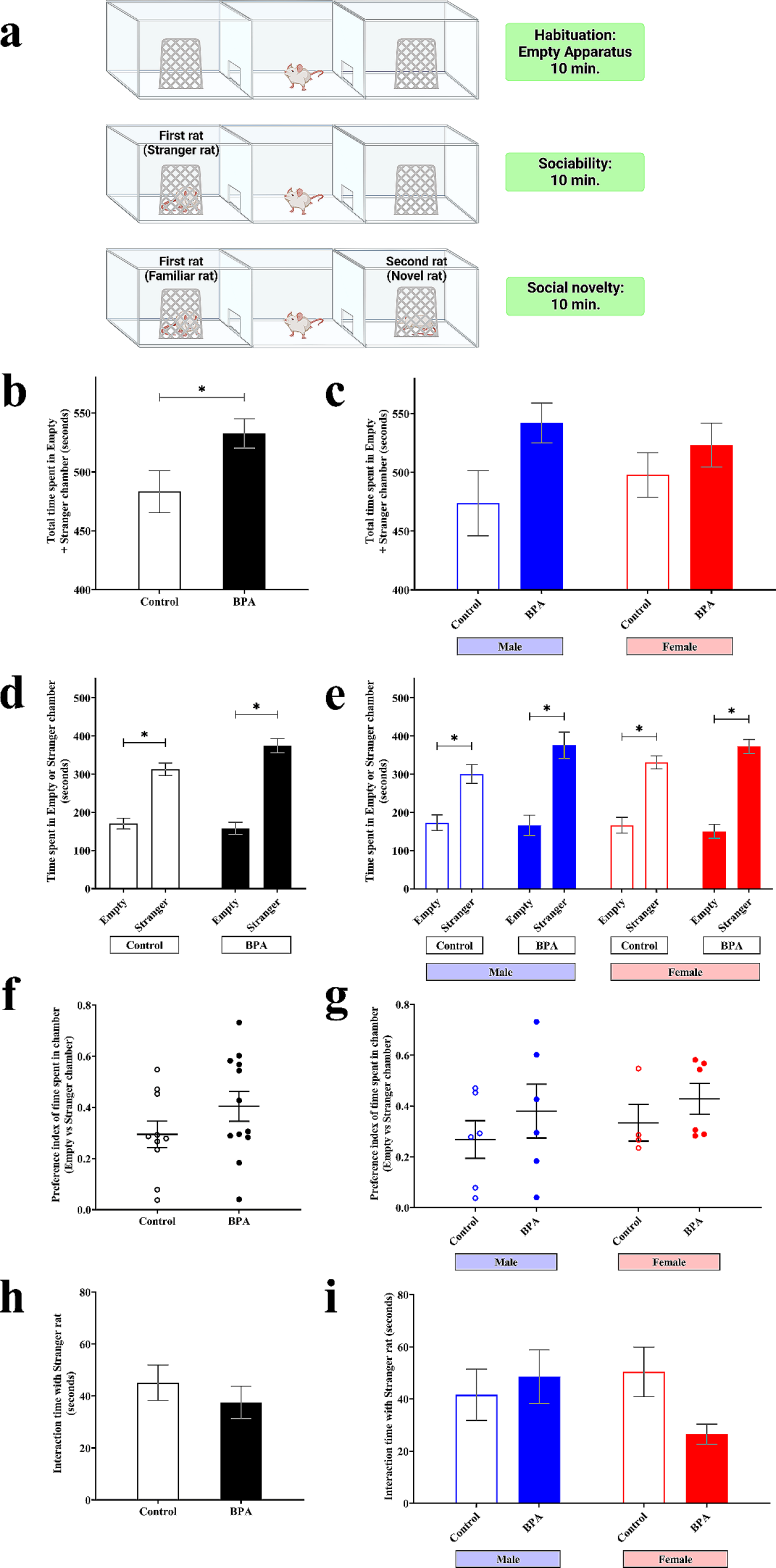
**Prenatal exposure to BPA did not affect sociability evaluated by the three-chamber test.** (**a**) The sociability and social novelty in the offspring were assessed using three-chamber tests. (**b, c**) For the sociability test, the total times that the rat spent in the empty chamber and chamber containing a stranger rat (treatment *F*_1,18_ = 4.546 and P value = 0.047, sex *F*_1,18_ = 0.014 and P value = 0.906, P value = 0.342 for treatment × sex, by two-way ANOVA), (**d, e**) the time that the rat spent in the empty chamber or chamber containing a stranger (treatment *F*_1,68_ = 1.808 and P value = 0.187, sex *F*_1,68_ = 0.006 and P value = 0.940, chamber *F*_1,68_ = 107.885 and P value < 0.001 and P value = 0.726 for treatment × sex × intruder, by three-way ANOVA), (**f, g**) preference index of time spent in the empty or stranger chamber were measured (treatment *F*_1,18_ = 1.515 and P value = 0.234, sex *F*_1,18_ = 0.464 and P value = 0.505, P value = 0.920 for treatment × sex, by two-way ANOVA), and (**h, i**) the times that the rat spent interacting (sniffing time) with a stranger (treatment *F*_1,18_ = 0.904 and P value = 0.354, sex *F*_1,18_ = 0.552 and P value = 0.467, P value = 0.101 for treatment × sex, by two-way ANOVA). Data are presented as the mean ± SEM. *P value < 0.05

**Fig. 5:**
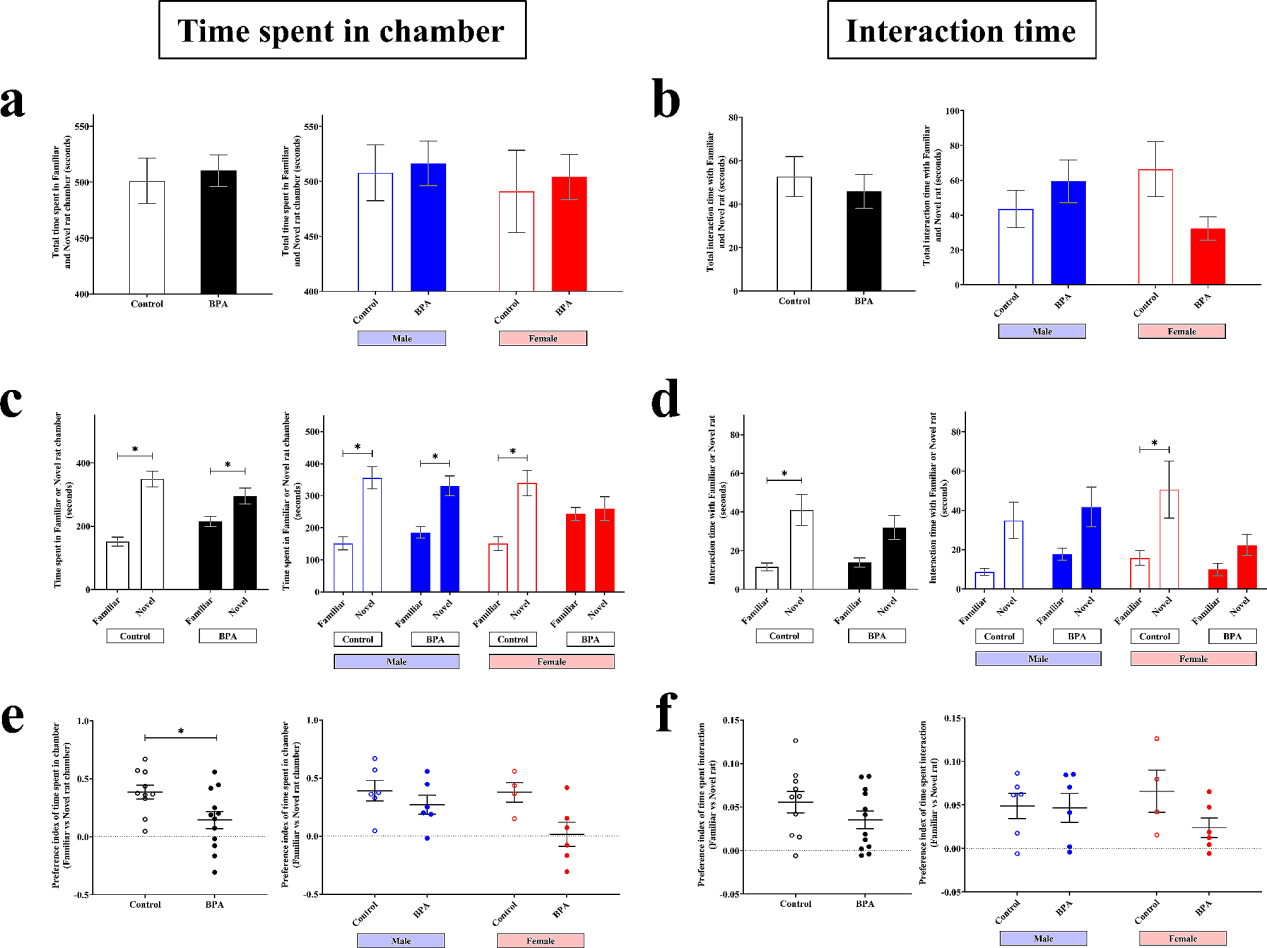
**Prenatal exposure to BPA impaired social novelty evaluated by the three-chamber test in a sex-dependent manner.** (**a**) The total times that the rat spent in the familiar and novel chamber (treatment *F*_1,18_ = 0.183 and P value = 0.674, sex *F*_1,18_ = 0.336 and P value = 0.570, P value = 0.993 for treatment × sex, by two-way ANOVA), (**b**) total time that the rat spent interacting with familiar and novel rat (treatment *F*_1,18_ = 0.648 and P value = 0.431, sex *F*_1,18_ = 0.038 and P value = 0.849, P value = 0.040 for treatment × sex, by two-way ANOVA), (**c**) the time that the rat spent in the familiar chamber or novel chamber (treatment *F*_1,68_ = 0.069 and P value = 0.795, sex *F*_1,68_ = 0.126 and P value = 0.724, chamber *F*_1,68_ = 45.260 and P value < 0.001 and P value = 0.182 for treatment × sex × intruder, by three-way ANOVA), (**d**) The time that the rat spent interacting with familiar or novel rat (treatment *F*_1,68_ = 0.797 and P value = 0.378, sex *F*_1,68_ = 0.046 and P value = 0.831, chamber *F*_1,68_ = 22.845 and P value < 0.001 and P value = 0.328 for treatment × sex × intruder, by three-way ANOVA), (**e**) the preference index of time spent in the familiar or novel chamber (treatment *F*_1,18_ = 6.494 and P value = 0.020, sex *F*_1,18_ = 2.022 and P value = 0.172, P value = 0.219 for treatment × sex, by two-way ANOVA), and (**f**) the preference index of time that the rat spent interacting with familiar and novel rat were measured (treatment *F*_1,18_ = 1.876 and P value = 0.188, sex *F*_1,18_ = 0.030 and P value = 0.863, P value = 0.232 for treatment × sex, by two-way ANOVA). Data are presented as the mean ± SEM. *P value < 0.05

We further performed the three-chamber test, which allowed us to examine the sociability and social novelty of rat pups prenatally exposed to BPA (*n* = 12, 6 males and 6 females) or vehicle control (*n* = 10, 6 males and 4 females) (Figs. 4 and 5). Sociability was used to determine the ability of the subject offspring to approach another rat staying on one side of the three-chamber while another had no rats. The schematic diagram of this test is shown in Fig. 4a. We found that the total time spent in both empty chamber and stranger chamber was significantly increased in offspring prenatally exposed to BPA compared to control offspring (Fig. 4b), indicating that prenatal BPA exposure may cause hyperactivity, which is a comorbid condition frequently reported in ASD cases [100]. By comparing between time spent in the stranger chamber and time spent in the empty chamber, we found that, like the vehicle control group, male and female offspring prenatally exposed to BPA spent more time in the stranger chamber than in the empty chamber (Fig. 4d and e). Moreover, the time that BPA-treated rats interacted with stranger rats was also assessed (Fig. 4h and i). We found that female offspring prenatally exposed to BPA, but not males, tended to spend less time interacting with the stranger rat compared to the sex-matched control (Fig. 4h and i). These findings suggest that prenatal BPA exposure may cause hyperactivity in both sexes of the offspring and impair the sociability of female offspring but did not affect those of the male offspring.

Next, we further determined the social novelty of the rat offspring prenatally exposed to BPA or vehicle control. After the subject offspring encountered the first rat (familiar rat), we introduced the second rat (stranger rat) to another side of the three-chamber apparatus. We allowed the subject rat to freely explore the familiar and stranger rats (Fig. 5a). We measured the time that the subject offspring stayed in each chamber and spent interacting with the familiar or novel rat. The results showed that there was no significant difference in the total time spent in the chamber containing both familiar and novel rats between prenatal BPA exposure offspring and control offspring in both males and females (Fig. 5a). When looking into each side of the chamber, both male offspring prenatally exposed to BPA and male control offspring spent significantly more time in the chamber containing novel rats than the familiar rat (Fig. 5c, right). In addition, female control offspring also spent significantly more time in the chamber containing the novel rat than the familiar rat (Fig. 5c, right). However, female offspring prenatally exposed to BPA did not show a significant difference between the time spent in the chamber containing novel and familiar rats (Fig. 5c, right). We also measured the time that the subject offspring interacted with the familiar and novel rats. The results were consistent with the two-trial result that female offspring prenatally exposed to BPA did not show a difference in time spent interacting with familiar and novel rats (Fig. 5d). In addition, the total interaction time with both familiar and novel rats of prenatal BPA exposure female but not male offspring tended to decrease compared with control offspring. (Fig. 5b). Moreover, the preference index, calculated by the differences in time spent between the novel and familiar chamber divided by total time spent in the novel and familiar chamber, significantly decreased in the BPA group compared to the control (Fig. 5e, left). Interestingly, the preference index of female offspring prenatally exposed to BPA, but not males, tended to decrease compared to the control group (Fig. 5e, right). These results suggest that prenatal BPA exposure impairs the social novelty behavior of female offspring but not males.

### Associations between BPA-responsive genes and disrupted neurological functions

**Fig. 6:**
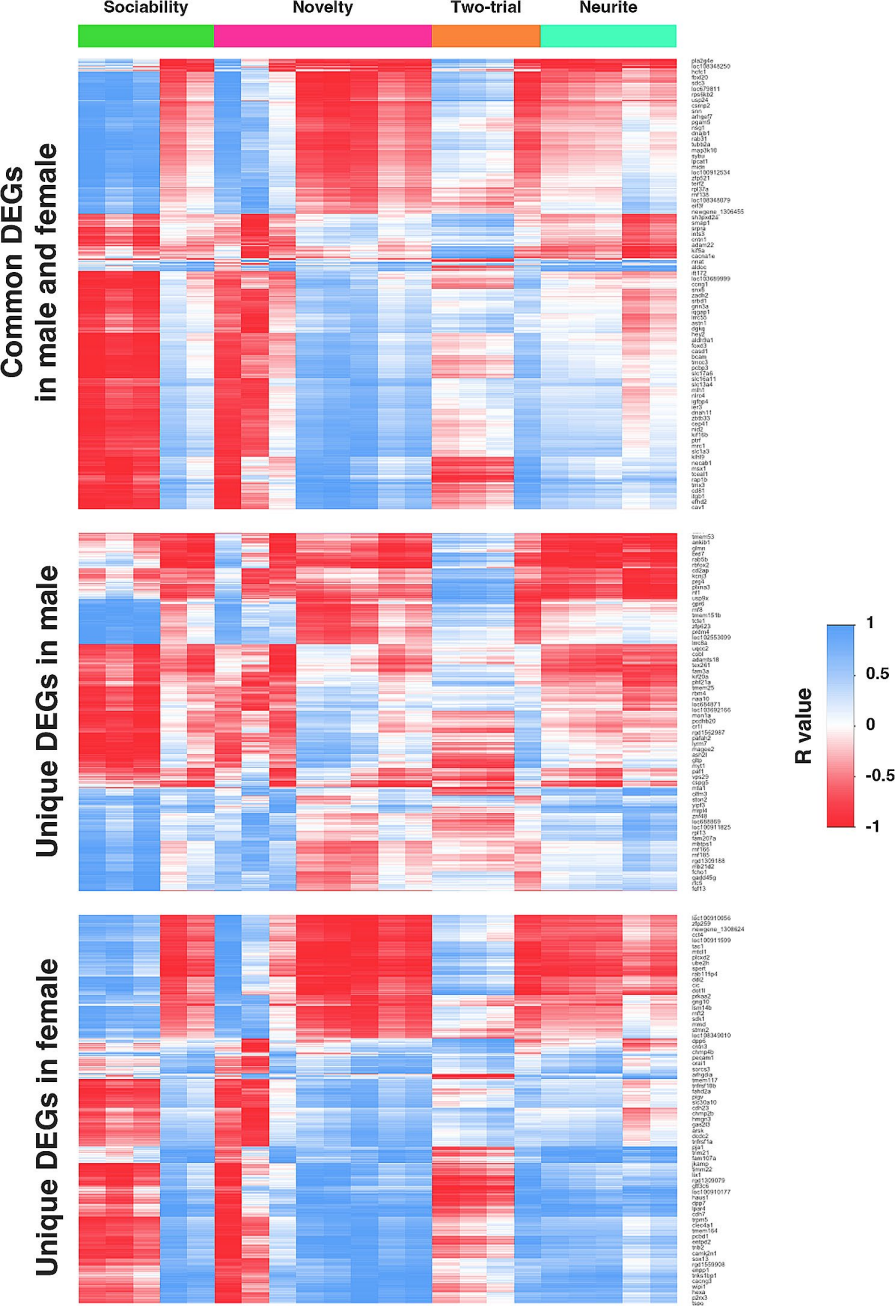
**Heatmap of the correlation matrix between DEGs’ expression levels and neurological phenotypes.** The correlation heatmap illustrates the relationships between the expression levels of DEGs that are unique to males, unique to females, and common to both genders, as well as their associations with neurological functions. The color scale represents the correlation coefficient values, ranging from red (indicating an inverse correlation) to blue (indicating a positive correlation)

**Fig. 7:**
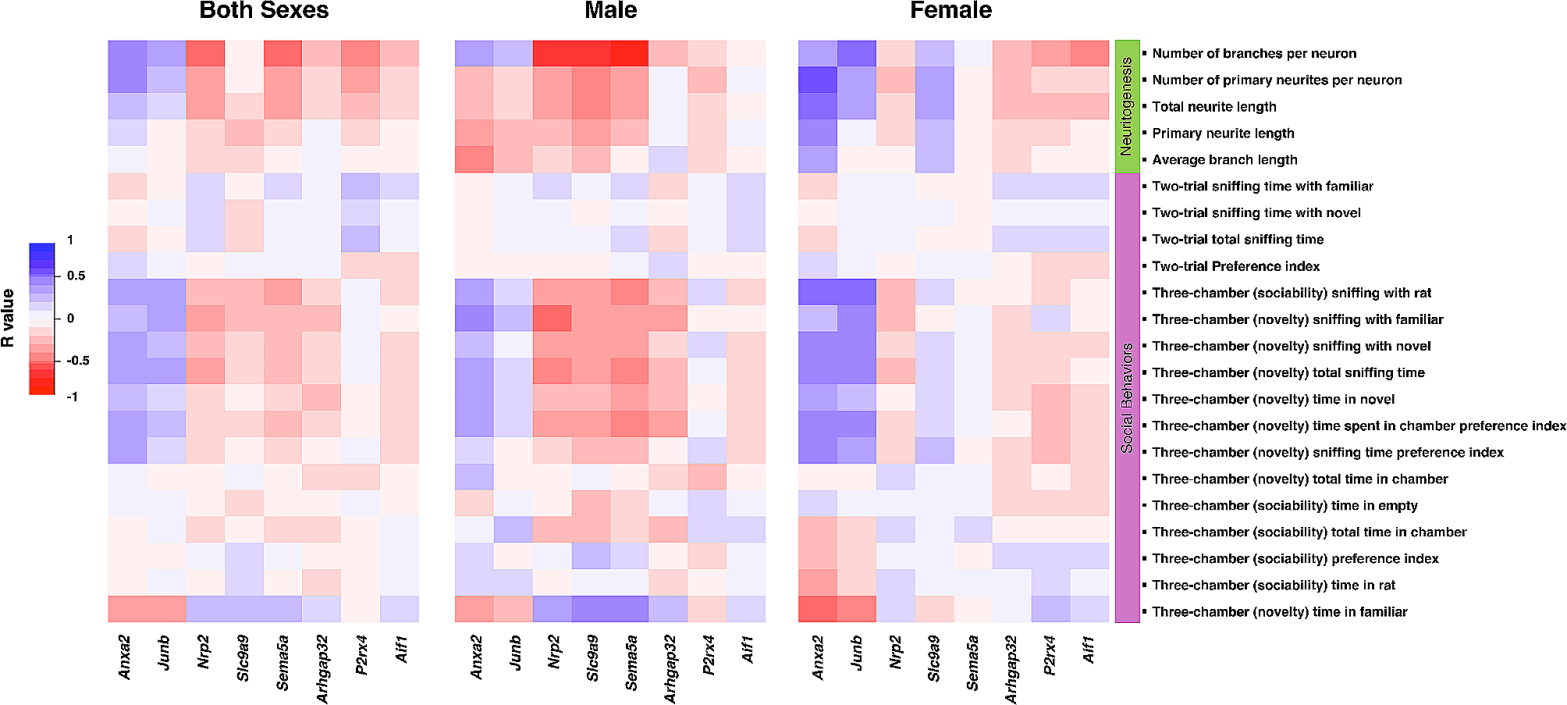
**Heatmap of the correlation matrix between gene expression levels and neurological phenotypes.** Correlation heatmap of the correlations between the expression levels of *Anxa2*, *Junb*, *Nrp2*, *Slc9a9*, *Sema5a*, *Arhgap32*, *P2rx4*, and *Aif1* determined by qRT-PCR and neurological functions. The color scale denotes the correlation coefficient value from red (inverse correlation) to blue (positive correlation)

To investigate whether changes in gene expression due to prenatal BPA exposure might contribute to alterations in neuritogenesis and social behaviors, we compared the differentially expressed genes (DEGs) identified in our previous RNA-seq analysis of prefrontal cortex tissues from neonatal rat pups exposed to either BPA or vehicle control with the neurological traits assessed in this study. The neurological parameters included various measures from the three-chamber test (sociability and social novelty), the two-trial test, and neuritogenesis-related metrics, as detailed in Fig. 6. Using Pavlidis Template Matching (PTM) analysis (P value < 0.05), we identified 3702 out of the 6644 DEGs responsive to prenatal BPA exposure that exhibited significant changes correlated with at least one neurological trait. Of these, 438 DEGs were correlated with neuritogenesis, and 3469 DEGs were correlated with social behaviors. Furthermore, we analyzed DEGs unique to males, females, or shared to both (Additional file 5) to explore their correlation with neurological traits (Fig. 6). Notably, the heatmap displaying the correlation values between DEGs within each group and neurological traits revealed a sex-specific pattern, suggesting that distinct sets of genes may regulate these neurological traits in a sex-specific manner. Detailed lists of DEGs whose expression levels correlated with neurological characteristics can be found in Additional file 9. In addition to the DEGs identified in response to BPA exposure from our previous RNA-seq analysis, we specifically examined the association between selected genes related to neuritogenesis and social behaviors (i.e., *Anxa2*, *Junb*, *Nrp2*, *Slc9a9*, *Sema5a*, *P2rx4*, *Arhgap32*, and *Aif1*) and changes in neuritogenesis and social behaviors (Fig. 7 and Additional file 10). Interestingly, our findings revealed that certain neurological traits exhibited sex-dependent correlations with the expression of these selected genes. For instance, the expression of *Nrp2*, *Slc9a9*, and *Sema5a* exhibited a robust inverse correlation with neuritogenesis, particularly the number of branches of prefrontal cortical neurons, in males (R value = − 0.800, − 0.760, − 0.835, respectively; Additional file 10) but not in females (R value = − 0.127, 0.262, 0.099, respectively; Additional file 10). Besides these genes, the expression of *Anxa2* and *Junb* also showed a female-specific positive correlation with neuritogenesis and social behavior functions, including the number of primary neurites (*Anxa* R value = 0.626), neuronal branches (*Junb* R value = 0.591), total neurite length (*Anxa* R value = 0.547), sociability sniffing time with rat (*Anxa* R value = 0.508, *Junb* R value = 0.502), social novelty time sniffing novel rat (*Anxa* R value = 0.491) and total sniffing time (*Anxa* R value = 0.493) (Additional file 10). These results suggest that BPA may exert its effects on neuritogenesis and social behaviors in offspring through sex-specific molecular mechanisms, and these genes may play a crucial role in the sex differences in the effects of prenatal BPA exposure on neuritogenesis and social behaviors.

### The effects of *Sema5a* suppression on male cortical development

**Fig. 8:**
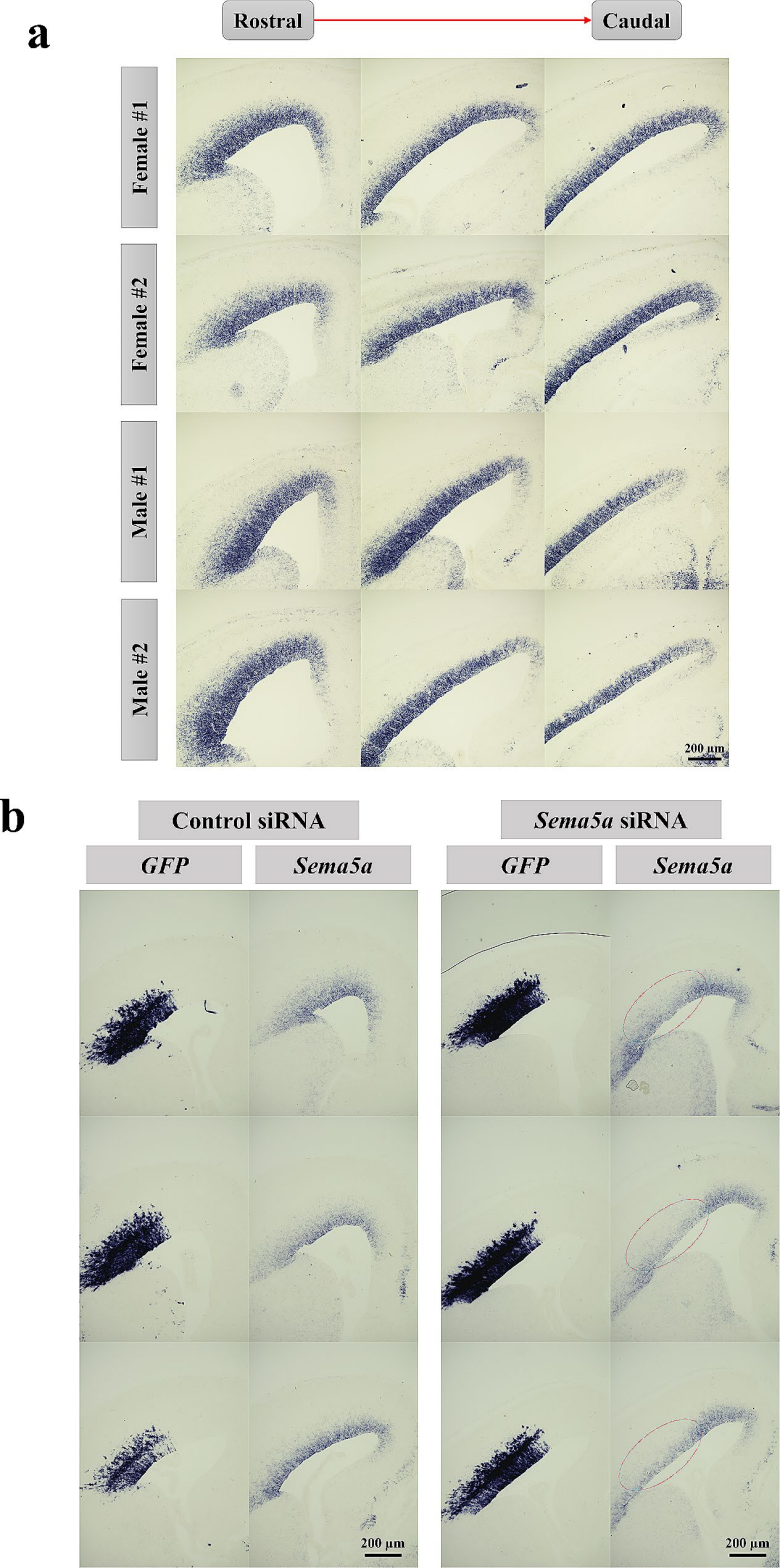
**In situ hybridization (ISH) analyses using E14.5 embryonic mouse brain sections.** (**a**) ISH results revealed the expression of *Sema5a* in the cortical primordium of E14.5 mouse brain (**b**) and the efficiency of control siRNA and *Sema5a* siRNA (red circle) after performing in utero electroporation, which could be observed in the same position as the area expressing GFP

Previous qRT-PCR results showed that prenatal BPA exposure caused the significant downregulation of *Sema5a* in the prefrontal cortex of male offspring prenatally exposed to BPA compared with control, but not in females. In addition, previous studies have revealed that the dysregulation of *Sema5a* causes aberrant synaptogenesis in the hippocampus and cognitive deficits [101, 102]. However, it is still unknown whether the male-specific downregulation of *Sema5a* mediated by prenatal BPA exposure could disrupt male cortical development. To investigate the effects of *Sema5a* suppression on the male developing cortex, siRNA-mediated knockdown of *Sema5a* using in utero electroporation technique was performed in mice. As Sema5a proteins in rats and mice are orthologs and the protein sequences are highly conserved with more than 98.5% sequence similarities (Additional file 11), which strongly suggests that they share the same functions between species, and siRNA-mediated knockdown using in utero electroporation technique is more feasible in mice, we used male mice as a model to investigate the effects of *Sema5a* suppression on male cortical development. We determined the *Sema5a* gene expression in the mice’s embryonic cortical primordium at embryonic day 14.5 (E14.5) using an in situ hybridization technique. We found that *Sema5a* mRNA was abundantly expressed at the ventricular zone (VZ) of the telencephalon of E14.5 mice embryos (Fig. 8a), indicating that it might play a crucial role in cortical development. We further determined the function of *Sema5a* during cortical development by using RNA interference to knockdown *Sema5a* expression. In this study, we used in utero electroporation technique to deliver double-stranded RNA molecule (siRNA) and green fluorescence protein plasmid (*pCAG-EGFP*) to progenitor cells located at the VZ of the E14.5 embryo brain. We first determined the efficiency of *Sema5a* siRNA. We found that it can downregulate the *Sema5a* in the mice’s embryonic cortical primordium at E14.5 (Fig. 8b). The electroporated brain sections were immunofluorescence stained with anti-GFP and other markers and imaged using confocal microscopy. Then, we equally divided the cortical region into 10 layers (10 bins) in E18.5 brains and counted the GFP^+^ cells.

**Fig. 9:**
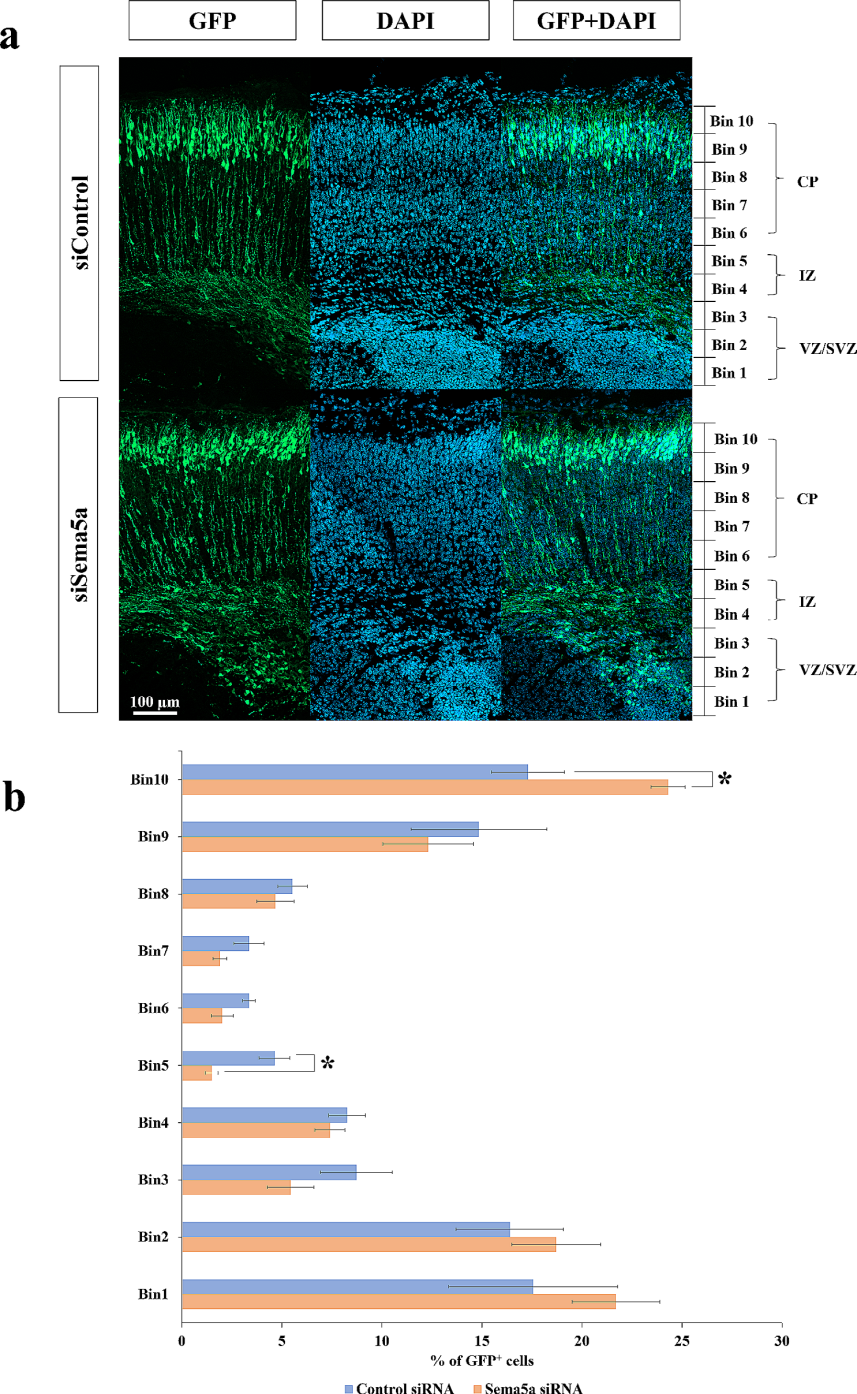
***Sema5a*'s role in neuronal migration during corticogenesis when in utero knockdown from E14.5-E18.5.** (**a**) Representative images of immunofluorescence staining of anti-GFP sections in utero electroporated by control siRNA or *Sema5a* siRNA. (**b**) The percentage of GFP^+^ cells in each bin from the VZ (bin 1) to the CP (bin 10) at E18.5 after the knockdown of *Sema5a* at E14.5. Data are presented as the mean ± SEM. *P value < 0.05. *Indicates a statistically significant difference between the knockdown and the control groups. CP: cortical plate; IZ: intermediate zone; SVZ: subventricular zone; VZ: ventricular zone

**Fig. 10:**
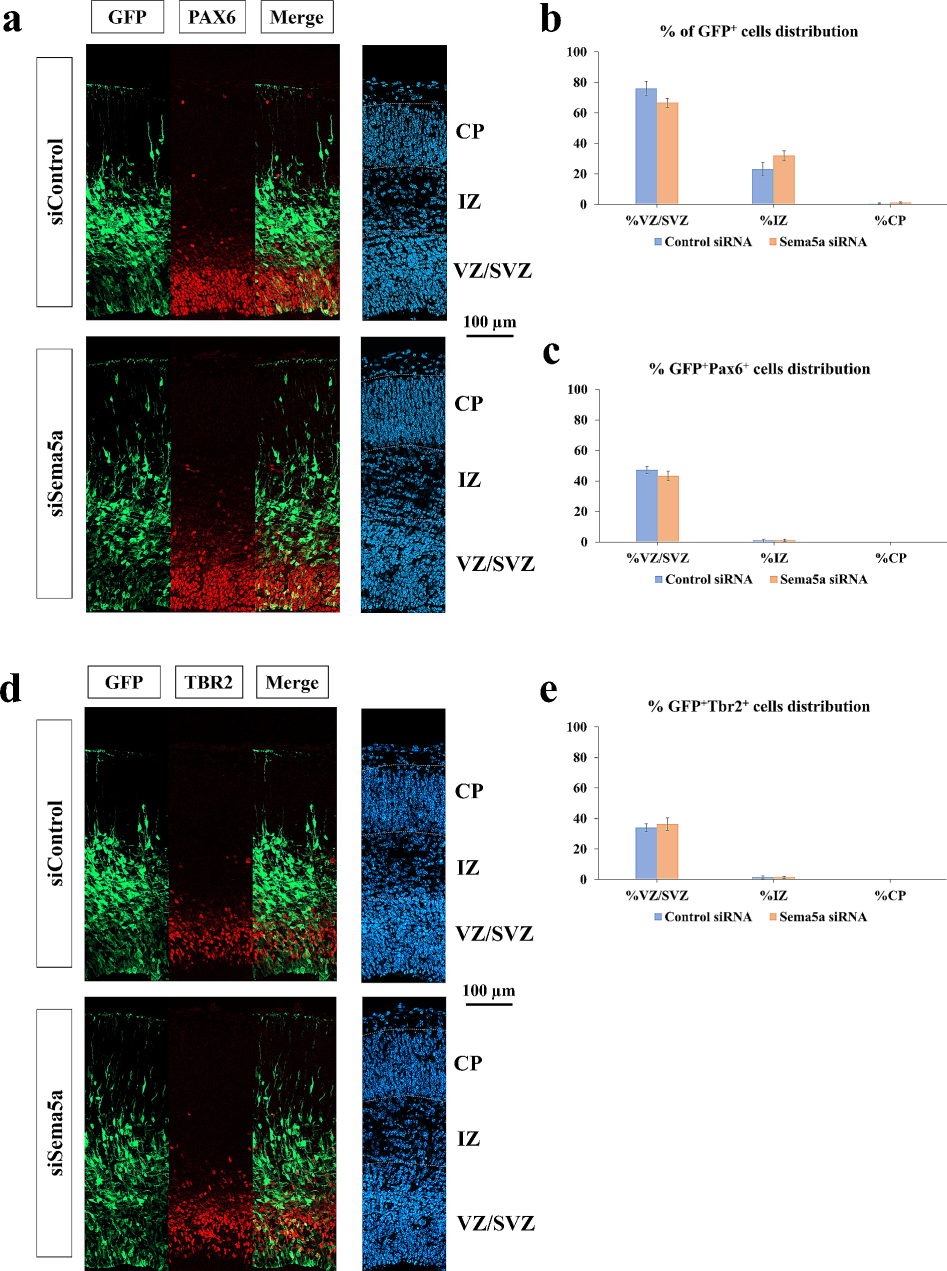
**Effects of downregulated *Sema5a* from E14.5-E16.5 on the number of Pax6**^**+**^** cells and Tbr2**^**+**^** cells.** (**a**) Representative images of immunofluorescence staining of anti-GFP and anti-Pax6 sections in utero electroporated by control siRNA or *Sema5a* siRNA. (**b**) The percentage of GFP^+^ cells in the VZ/SVZ, IZ, and CP at E16.5 after the knockdown of *Sema5a* at E14.5. (**c**) The percentage of GFP^+^/Pax6^+^ cells in the VZ/SVZ, IZ, and CP at E16.5 after knockdown of *Sema5a* at E14.5. (**d**) Representative images of immunofluorescence staining of anti-GFP and anti-Tbr2 sections in utero electroporated by control siRNA or *Sema5a* siRNA. (**e**) The percentage of GFP^+^/Tbr2^+^ cells in the VZ/SVZ, IZ, and CP at E16.5 after the knockdown of *Sema5a* at E14.5. Data are presented as the mean ± SEM. *P value < 0.05. *Indicates a statistically significant difference between the knockdown and the control groups. CP: cortical plate; IZ: intermediate zone; SVZ: subventricular zone; VZ: ventricular zone

Interestingly, transfected cells located in the upper layer of the cortical plate (CP) and located less in bin 5 corresponded to the upper intermediate zone (IZ) in *Sema5a*-knockdown samples compared with the control (Fig. 9a and b). Conversely, the percentage of GFP^+^ cells co-transfected with *Sema5a* siRNA was significantly increased in the most upper superficial layer (bin 10, layers II/III). On whether the knockdown of *Sema5a* interfered with the differentiation of progenitor to neuron transition, we analyzed the brains 2 days after the electroporation and counted the number of Pax6^+^ neural stem cells and Tbr2^+^ intermediate progenitors at E16.5 (Fig. 10a). The results showed no significant difference in the number of GFP^+^ (Fig. 10b), GFP^+^Pax6^+^ (Fig. 10c), and GFP^+^Tbr2^+^ cells (Fig. 10d and e) of the *Sema5a* knockdown condition compared with the control. Taken together, these results suggested that the downregulation of *Sema5a* could increase neuronal migration without affecting the differentiation process during embryonic development.

## Discussion

The sex-dependent associations between prenatal/postnatal BPA exposure and child behaviors associated with ASD have been reported in several studies, as mentioned earlier, albeit inconsistent [12, 103, 104]. Our recent studies have demonstrated that prenatal exposure to BPA altered the transcriptome-interactome profiles of genes associated with ASD in the hippocampus and the prefrontal cortex of neonatal rat offspring in a sex-dependent manner [36, 37]. In the offspring’s hippocampus, we found that BPA may exert its sex-specific effects by direct interactions with sex hormone receptors (i.e., Ar and Esr1) and other transcription factors, including Kdm5b, Smad4, and Tcf7l2, to regulate genes involved in synaptogenesis, neuronal viability, neuritogenesis, learning/memory [37–39]. In the offspring’s prefrontal cortex, we found that genes differentially expressed in response to prenatal exposure to BPA are transcriptional targets of Ar, Esr1, and Rora, all of which have been implicated in the sex bias of ASD [105–108]. Other ASD-related transcription factors, including Sox5, Tcf4, and Yy1, were also predicted to be potential targets of BPA in the prefrontal cortex [36]. However, the molecular mechanisms underlying the sex-dependent effects of BPA on neurological functions and ASD-related behaviors are still unclear.

**Fig. 11:**
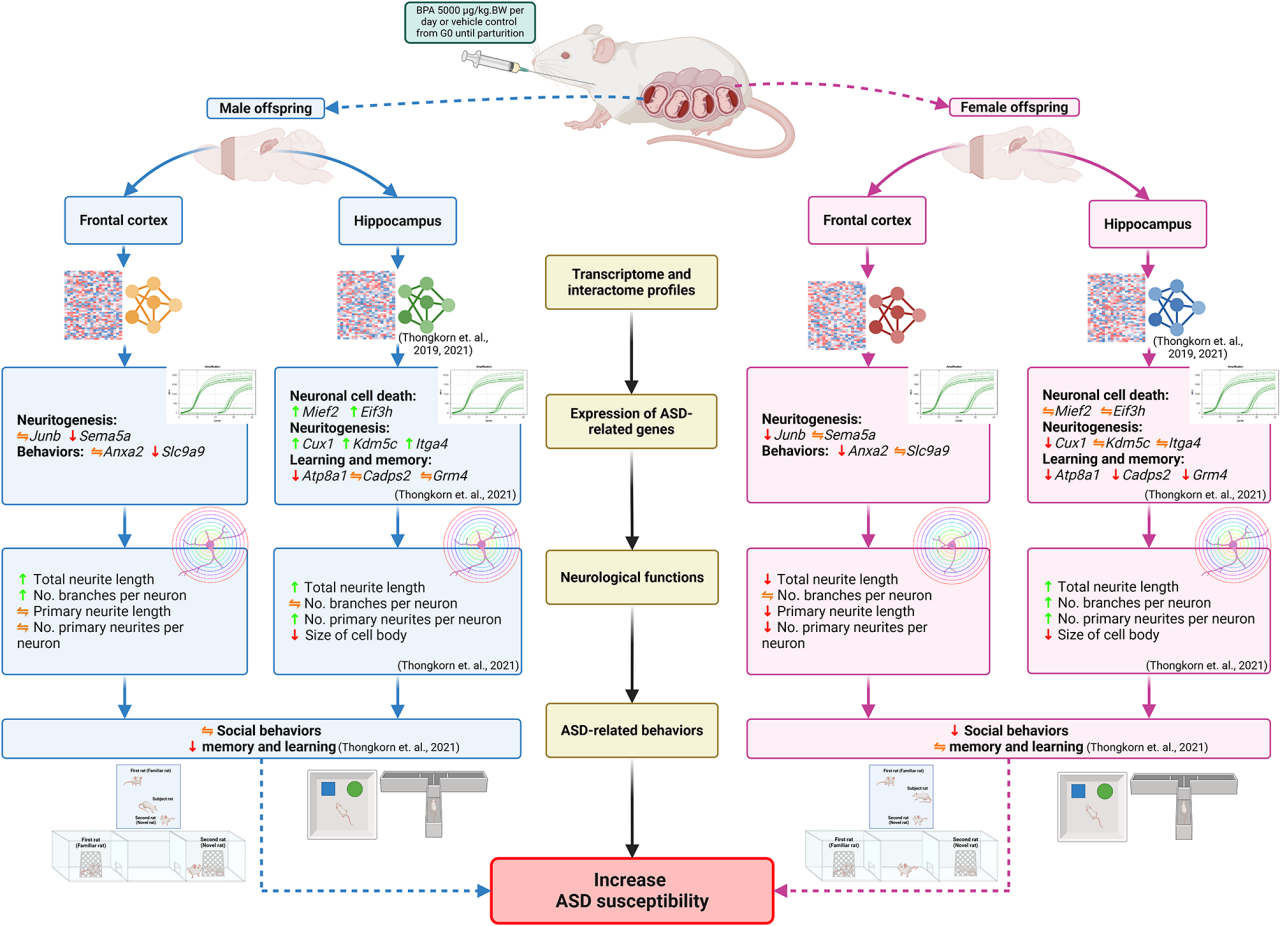
**A schematic diagram summarizing the main findings of this study.** This figure was created with BioRender.com (https://www.biorender.com/)

In this study, we are the first to demonstrate the sex differences in the effects of prenatal BPA exposure on transcriptome profiles of genes associated with neuritogenesis and ASD-related behaviors in the offspring’s prefrontal cortex. The significant findings of our study are summarized in Fig. 11. We showed that prenatal BPA exposure can exhibit both male-specific and female-specific effects depending on neurological functions and behaviors (Fig. 11). Male-specific effects of prenatal exposure to BPA included suppressing *Sema5a*, *Nrp2*, and *Slc9a9* expression and promoting neurite formation and branching in the prefrontal cortex. On the other hand, female-specific effects included downregulating *Anxa2* and *Junb* expression, reducing neurite formation and branching, and causing impaired social novelty.

*Sema5a* encodes semaphorin 5A, which is a membrane protein containing a semaphorin domain and several thrombospondin type-1 repeats [109]. Members of the semaphorin family are involved in axonal guidance during neural development. This gene has been implicated as an autism susceptibility gene [110, 111]. Mosca-Boidron et al. (2016) performed genetic analyses and found the partial deletion of *SEMA5A* in ASD children [111]. Moreover, specific genetic polymorphisms of *SEMA5A* are also strongly correlated with human hippocampal structure and function [112]. However, the role of *Sema5a* during corticogenesis was unclear. In this study, we revealed that prenatal exposure to BPA suppressed *Sema5a* expression only in the prefrontal cortex of male offspring but not in females (Fig. 1). We also further demonstrated that siRNA-mediated knockdown of *Sema5a* caused an abnormal increase in neuronal migration in the male developing cortex, leading to an increased number of neuronal cells in the uppermost layer of the cortical plate (Fig. 9). It is noteworthy that an increased number of neurons and forming of the gyrus of the frontal lobe were observed in the prefrontal cortex of people with ASD [113]. Moreover, the medial prefrontal cortex regions of individuals with ASD were also found to be abnormally enlarged [114]. This suggests that male-specific reduction of *Sema5a* may lead to abnormally increased neuritogenesis and cortical development reminiscent of ASD. Inasmuch as the downregulation of *Sema5a* was observed only in male offspring prenatally exposed to BPA, we, therefore, focused on the effects of *Sema5a* reduction on cortical development only in the male developing cortex in the siRNA-mediated knockdown assays in this study. However, future studies may include an investigation of the effects of *Sema5a* suppression in the female developing cortex as they might be different from the males.

*Slc9a9* gene encodes Solute Carrier Family 9 Member A9, which is a sodium/hydrogen exchanger (NHE) protein located on the membrane of late-recycling endosomes. It regulates the pH and maintains the cation homeostasis of endosomes [89]. Mutations in this gene have been associated with autism and attention-deficit/hyperactivity disorder [115]. A recent study has shown that *Slc9a9* knock-out (KO) mice lacked a preference for social novelty but did not show deficits in social approach [89], similar to what we observed in the offspring prenatally exposed to BPA, whose *Slc9A9* expression was significantly reduced in this study (Fig. 1d left, Fig. 5e, left). Yang et al. (2016) have also reported that mice lacking Slc9a9 emitted fewer calls and had shorter call durations, which suggested communication impairment. They also spent more time self-grooming, an indicator for restricted and repetitive behavior [89]. It is also noteworthy that, in this study, we found that rat offspring prenatally exposed to BPA spent more total time in the empty and stranger chambers than control (Fig. 4b), reminiscent of hyperactivity. This evidence suggests that prenatal BPA exposure may impair social novelty behavior and increase the risk of hyperactivity in offspring through the suppression of *Slc9a9*.

Another gene found to be downregulated in male offspring prenatally exposed to BPA is *Nrp2*. *Nrp2* encodes Neuropilin-2, which plays a significant role in neuritogenesis [116]. Neuropilin-2 is a transmembrane receptor protein that binds to various ligands, such as semaphorins and vascular endothelial growth factor (VEGF) [117]. These ligands are involved in guiding axon growth and neuronal development [80, 81]. Studies showed that coculture of nerve cells obtained from rat embryos with cell expression Sema III in the presence of Nrp2 antibody caused neurite outgrowth to move toward Sema III expressing cells, suggesting the role of *Nrp2* in regulating neuritogenesis [81]. This evidence suggests that the male-specific reduction of *Nrp2* in offspring prenatally exposed to BPA may be partly involved in increased neuritogenesis.

*Anxa2* encodes Annexin A2 protein, which is a member of the calcium-dependent phospholipid-binding protein family and is involved in the regulation of cellular growth and signal transduction pathways [118]. Inhibition of Annexin A2 in PC-12 cells resulted in a decreased ability to develop neurites during differentiation [119]. Moreover, annexin A2 was found to be necessary for the survival and neurite outgrowth of developing cortical neurons and the survival of glial cells [120]. Interestingly, a recent study has found that social isolation during adolescence resulted in abnormal locomotor, emotional, and cognitive behaviors and increased the expression of annexin A2 in the prefrontal cortex of adult rats [86]. These findings suggest that annexin A2 may be involved in the reduction of neurite length and branching, as well as impairment in social novelty, observed in female offspring prenatally exposed to BPA.

*Junb* encodes a protein member of the AP-1 family of transcription factors and plays a crucial role in regulating gene expression and has been implicated in various biological processes, including cell proliferation, differentiation, and apoptosis [121]. Several studies have shown that it is involved in several neurological functions, including neurite outgrowth and behaviors, including social, cognition, learning, and memory. A previous study found an increasing in Junb expression in the hippocampus after experiencing context-dependent memory retrieval [122]. In addition, the female mouse was placed in a chamber with a sexually experienced male exhibited increasing in Junb in specific parts of the cortex and hippocampus [123], suggesting that Junb might mediate sexual-related social behaviors. This is consistent with our result, which revealed that *Junb* was significantly decreased in female offspring prenatally exposed to BPA, together with deficits in social behaviors. This might support our result that *Junb* might be involved in regulating social behaviors, which is one of the hallmarks of ASD. In addition, the dimerization of Jun and Junb caused increased neurite outgrowth, suggesting the role of Junb in neuritogenesis [85]. This result is also consistent with our results that neuritogenesis was disrupted in female offspring prenatally exposed to BPA. However, we did not find any direct evidence that the decrease expression of *Junb* causes ASD susceptibility, which deserves further study.

Correlation analysis of the expression of *Sema5a* and neurological phenotypes showed that *Sema5a* was correlated with specific sociability phenotypes and social novelty in the three-chamber test. However, several parameters did not correlate with the expression of *Sema5a*, indicating that *Sema5a* might not be involved in regulating social behaviors. Moreover, we found an inverse correlation between *Sema5a* expression and the number of branches per neuron in the neurite formation assay in males. Interestingly, we found that primary cortical cells from male offspring prenatally exposed to BPA exhibited a significant increase in the number of branches per neuron, while the expression of *Sema5a* in response to prenatal BPA exposure was significantly decreased compared with vehicle control. This result might suggest the novel role of *Sema5a* in regulating neurite formation in response to prenatal BPA exposure, which needs to be studied further.

### Perspectives and significance

In summary, our study proposes that maternal BPA exposure during gestation leads to alterations in the transcriptome profiles within the prefrontal cortex of offspring, impacting neurological functions associated with ASD through sex-specific mechanisms (Fig. 11). BPA disrupts the transcriptome profiles of genes linked to ASD within the prefrontal cortex, resulting in a decrease in *Sema5a*, and *Slc9a9*, genes associated with neuritogenesis in male offspring, and an increase in *Anxa2* and *Junb*, genes linked to neuritogenesis and social behaviors in female offspring. These changes in BPA-responsive genes may involve additional mechanisms, such as axon guidance signaling, sex hormone-related transcription factor signaling, and epigenetic processes, contributing to the altered neurological functions associated with ASD in a sex-dependent manner, particularly affecting neuritogenesis and social behaviors. Remarkably, the significant downregulation of *Sema5a* appears to play a role in mediating neuritogenesis and neuronal migration during cortical development, although it may not directly result in social behavior deficits in male offspring. Moreover, the BPA-mediated substantial downregulation of *Slc9a9* in offspring may potentially be involved in impaired social behaviors and hyperactivity, although further investigation is required to confirm this hypothesis. Conversely, the pronounced downregulation of *Anxa2* and *Junb* in female offspring may primarily influence the regulation of social behaviors rather than neuritogenesis, warranting additional research. This study significantly enhances our understanding of the sex-specific BPA-mediated mechanisms within the offspring’s prefrontal cortex. It offers valuable insights into the role of environmental factors, particularly endocrine-disrupting chemicals, in influencing ASD susceptibility. These findings hold promise for the development of future therapeutic targets aimed at addressing BPA-related ASD, potentially leading to improved outcomes for affected individuals.

### Electronic supplementary material

Below is the link to the electronic supplementary material.

Additional file 1: List of primers for qRT-PCR analyses.

Additional file 2: List of differentially expressed genes in the prefrontal cortex of rat pups prenatally exposed to BPA when both male and female pups were combined into one group for each treatment.

Additional file 3: List of differentially expressed genes in the prefrontal cortex of male rat pups prenatally exposed to BPA.

Additional file 4: List of differentially expressed genes in the prefrontal cortex of female rat pups prenatally exposed to BPA.

Additional file 5: Venn diagram illustrating the number of differentially expressed genes (DEGs) found exclusively in males, exclusively in females, and in both males and females in the prefrontal cortex of offspring prenatally exposed to BPA.

Additional file 6: An interactome network of BPA-responsive genes in the prefrontal cortex of offspring prenatally exposed to BPA when both male and female pups were combined into one group for each treatment predicted by IPA software revealed interactions with the neurological functions and disorders associated with ASD (colored; red = upregulation; green = downregulation).

Additional file 7: An interactome network of BPA-responsive genes in the prefrontal cortex of male offspring prenatally exposed to BPA predicted by IPA software revealed interactions with the neurological functions and disorders associated with ASD (colored; red = upregulation; green = downregulation).

Additional file 8: An interactome network of BPA-responsive genes in the prefrontal cortex of female offspring prenatally exposed to BPA predicted by IPA software revealed interactions with the neurological functions and disorders associated with ASD (colored; red = upregulation; green = downregulation).

Additional file 9: The list of genes differentially expressed in the offspring prefrontal cortex in response to prenatal BPA exposure exhibited changes in the expression levels correlated with the neurological phenotypes.

Additional file 10: The Pearson’s correlation coefficient value of qRT-PCR validated genes that are differentially expressed in the offspring prefrontal cortex in response to prenatal BPA exposure that exhibited the changes in the expression levels correlated with the neurological phenotypes.

Additional file 11: Protein sequence alignment of Sema5a in mouse (NP_033180.2, Mus musculus) and rat (NP_001101129.1, Rattus norvegicus) (Accessed on 10 Jan 2024).

## Data Availability

The transcriptome profiling data used in this study have been deposited in the NCBI GEO DataSets database (GSE229073).
