# Supplementary figures and images for: Sex-specific impacts of prenatal bisphenol A exposure on genes associated with cortical development, social behaviors, and autism in the offspring’s prefrontal cortex
Source: Biol Sex Differ. 2024 May 15;15:40. doi: 10.1186/s13293-024-00614-2 (PMC11094985; doi:10.1186/s13293-024-00614-2)

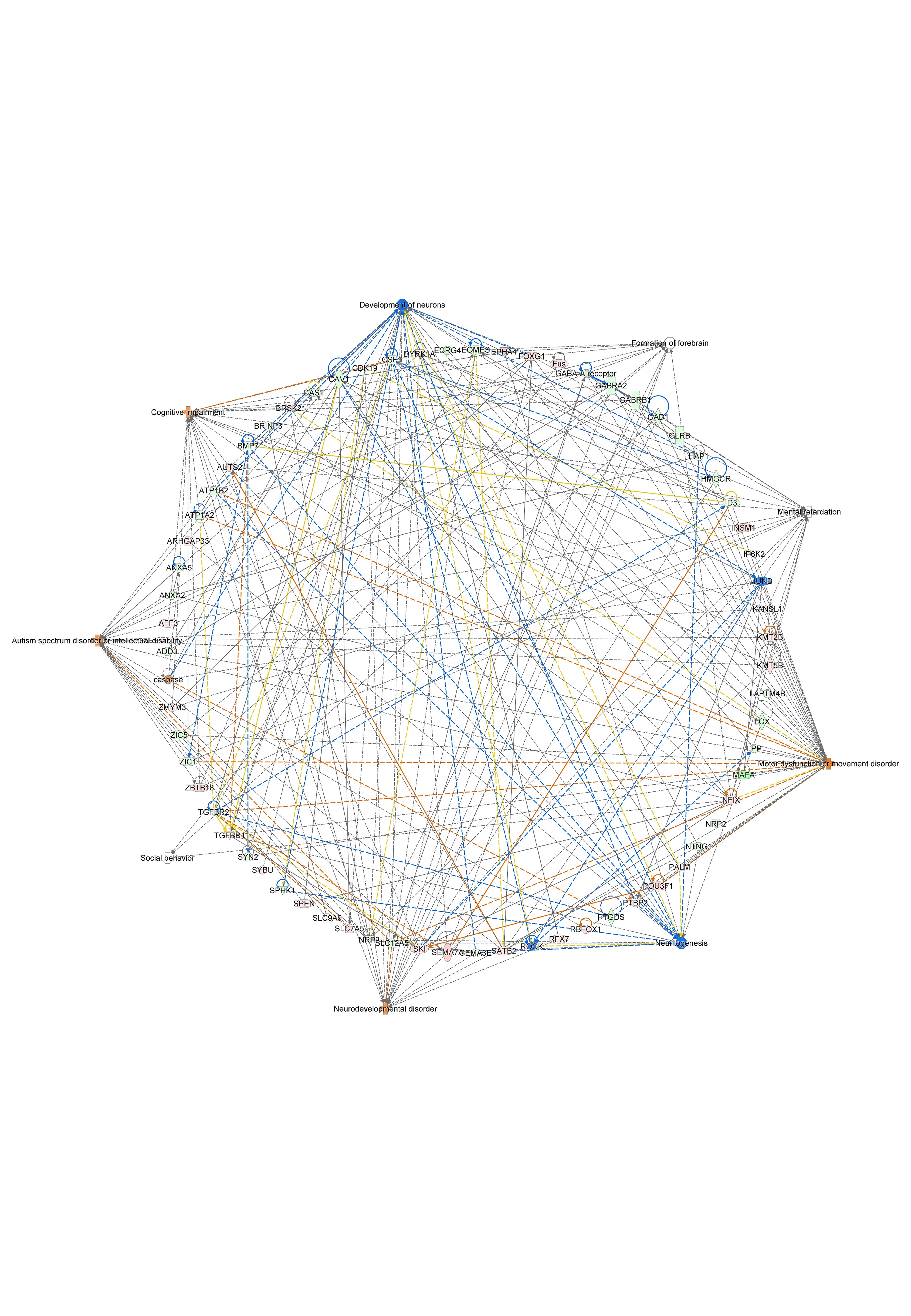

Supplement: Supplementary file 6 — Additional file 6: An interactome network of BPA-responsive genes in the prefrontal cortex of offspring prenatally exposed to BPA when both male and female pups were combined into one group for each treatment predicted by IPA software revealed interactions with the neurological functions and disorders associated with ASD (colored; red = upregulation; green = downregulation). [file 13293_2024_614_MOESM6_ESM.png]

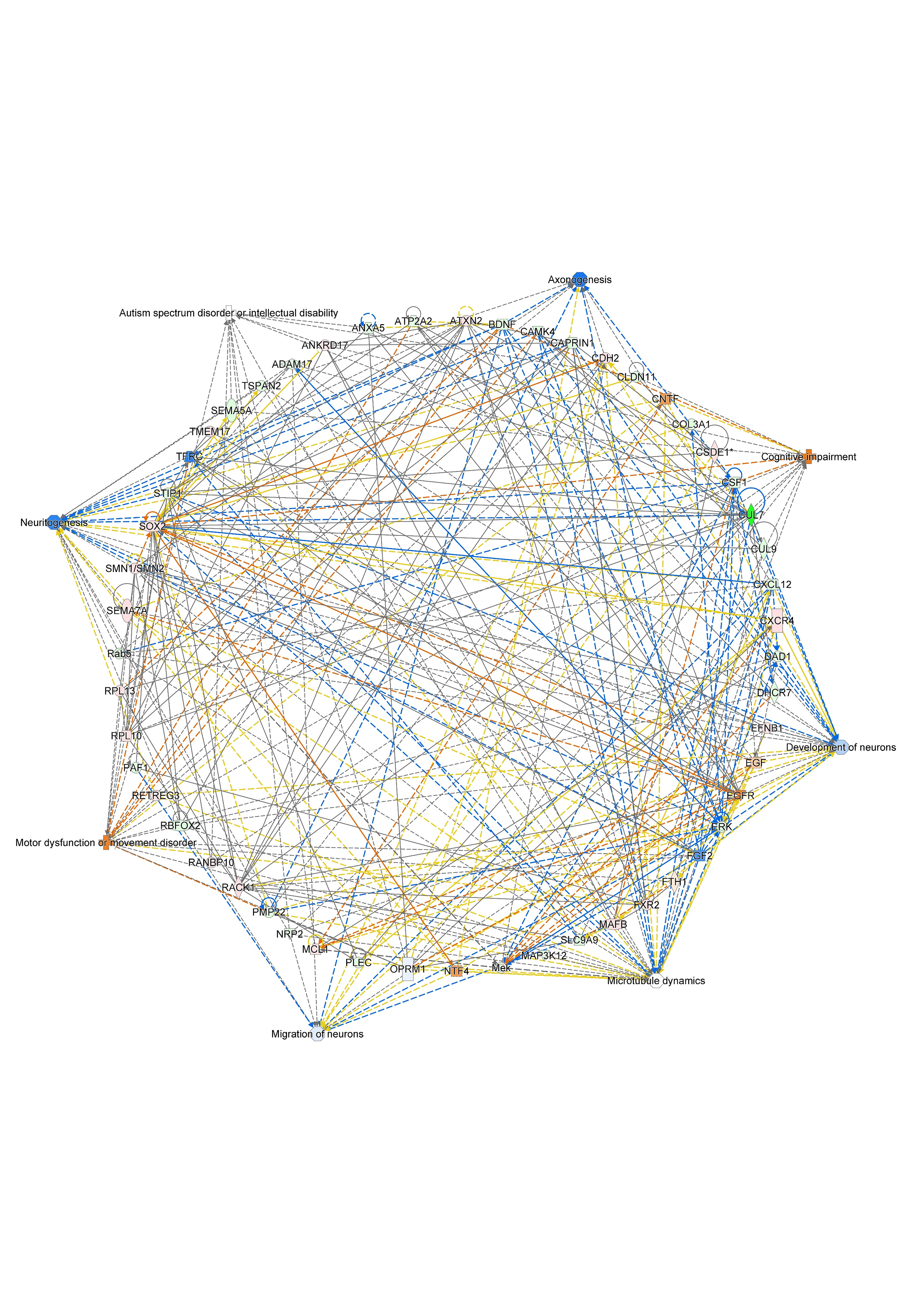

Supplement: Supplementary file 7 — Additional file 7: An interactome network of BPA-responsive genes in the prefrontal cortex of male offspring prenatally exposed to BPA predicted by IPA software revealed interactions with the neurological functions and disorders associated with ASD (colored; red = upregulation; green = downregulation). [file 13293_2024_614_MOESM7_ESM.png]

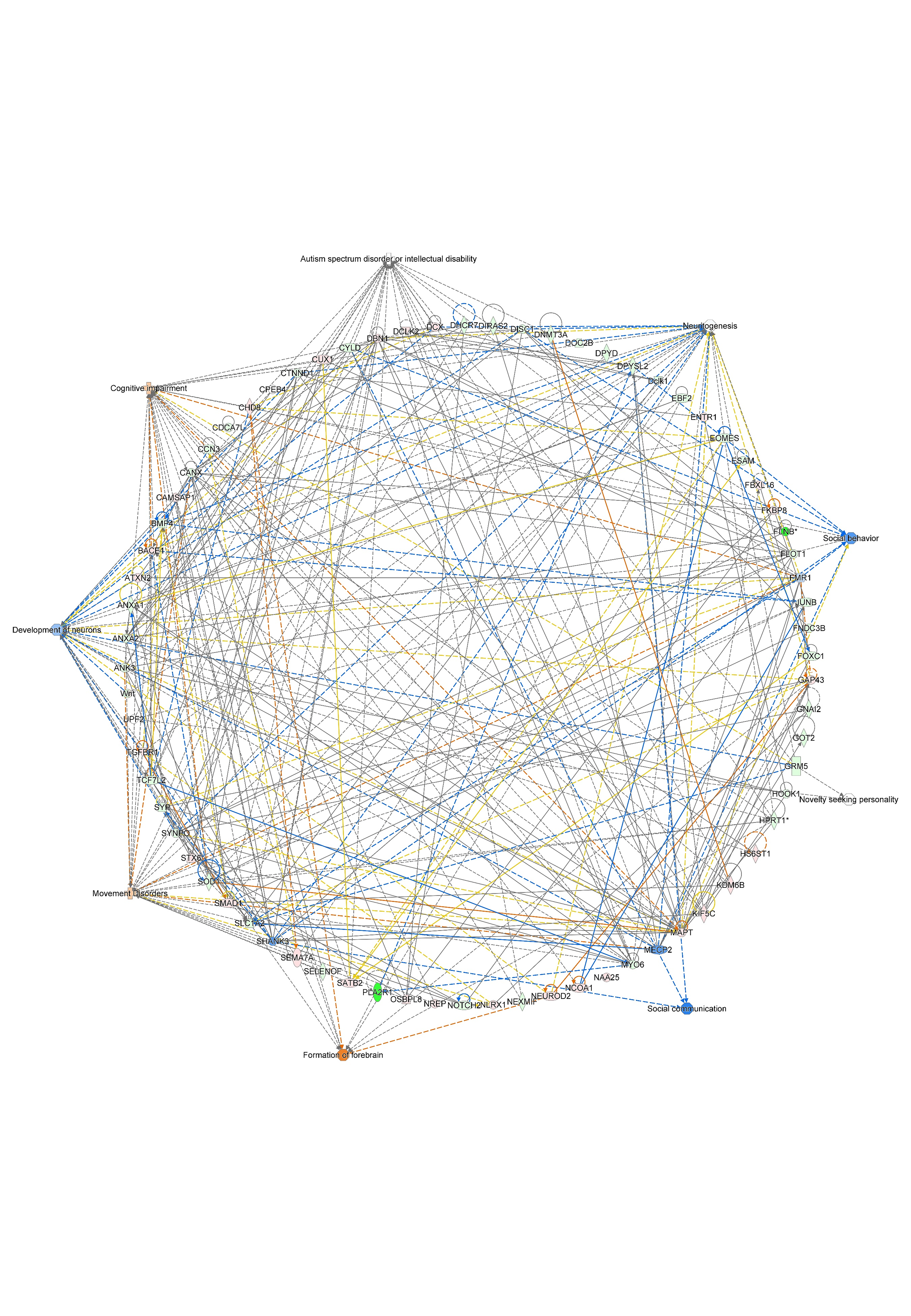

Supplement: Supplementary file 8 — Additional file 8: An interactome network of BPA-responsive genes in the prefrontal cortex of female offspring prenatally exposed to BPA predicted by IPA software revealed interactions with the neurological functions and disorders associated with ASD (colored; red = upregulation; green = downregulation). [file 13293_2024_614_MOESM8_ESM.png]

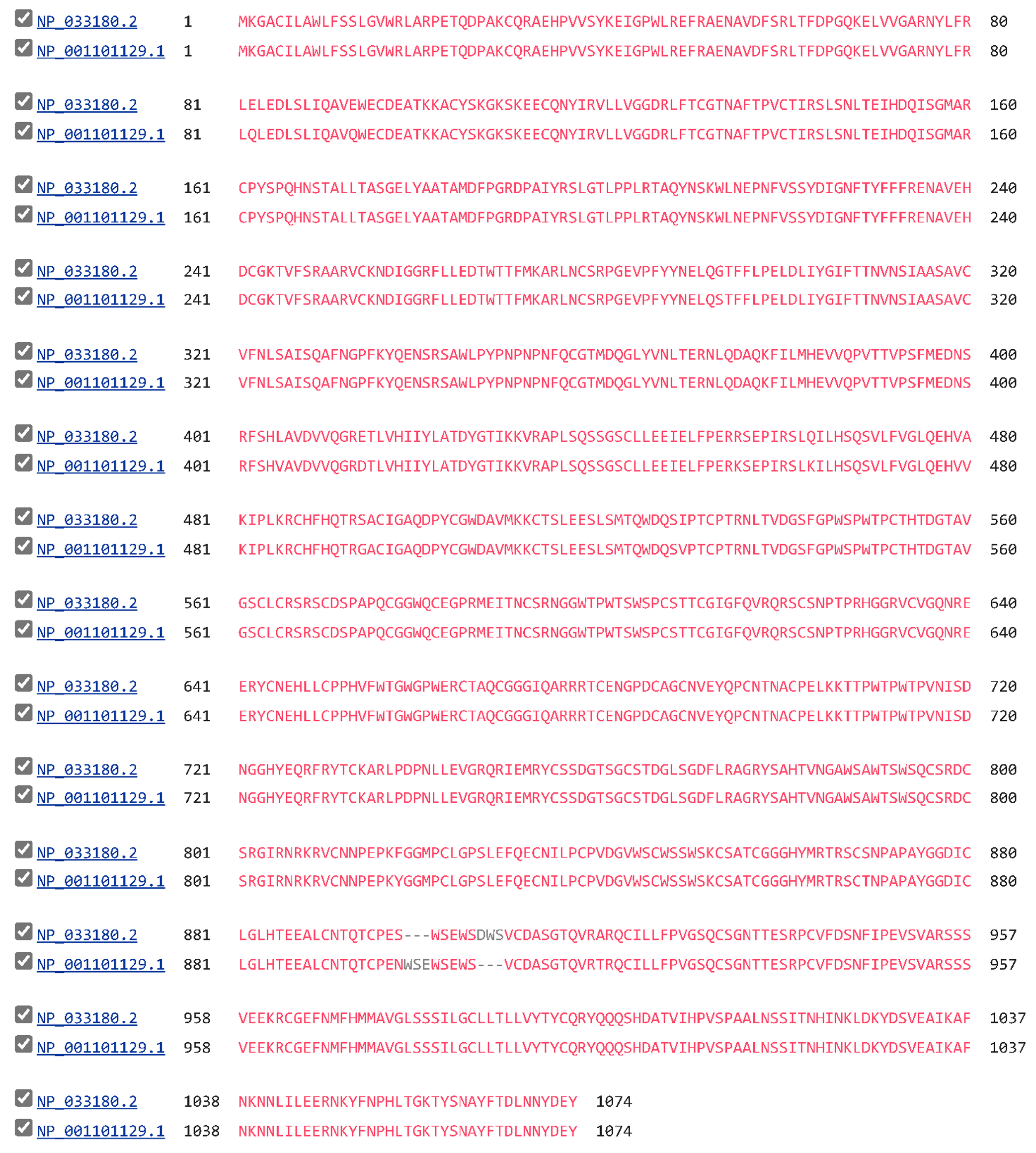

Supplement: Supplementary file 11 — Additional file 11: Protein sequence alignment of Sema5a in mouse (NP_033180.2, Mus musculus) and rat (NP_001101129.1, Rattus norvegicus) (Accessed on 10 Jan 2024). [file 13293_2024_614_MOESM11_ESM.png]
